# Entire CD3ε, δ, and γ humanized mouse to evaluate human CD3–mediated therapeutics

**DOI:** 10.1038/srep45839

**Published:** 2017-04-03

**Authors:** Otoya Ueda, Naoko A. Wada, Yasuko Kinoshita, Hiroshi Hino, Mami Kakefuda, Tsuneo Ito, Etsuko Fujii, Mizuho Noguchi, Kiyoharu Sato, Masahiro Morita, Hiromi Tateishi, Kaoru Matsumoto, Chisato Goto, Yosuke Kawase, Atsuhiko Kato, Kunihiro Hattori, Junichi Nezu, Takahiro Ishiguro, Kou-ichi Jishage

**Affiliations:** 1 Chugai Pharmaceutical Co., Ltd., Research Division, Fuji Gotemba Research Labs., 1-135, Komakado, Gotemba, Shizuoka, Japan; 2 Chugai Pharmaceutical Co., Ltd., Research Division, Kamakura Research Labs., 200, Kajiwara, Kamakura, Kanagawa, Japan; 3 Chugai Research Institute for Medical Science, Inc. 1-135, Komakado, Gotemba, Shizuoka, Japan; 4 Chugai Pharmabody Research Pte. Ltd., 3 Biopolis Drive, #07 - 11 to 16, Synapse, 138623, Singapore; 5 Chugai Pharmaceutical Co., Ltd., Translational Clinical Research Division, 1-1 Nihonbashi-Muromachi 2-Chome, Chuo-ku, Tokyo, Japan

## Abstract

T cell–mediated immunotherapy is an attractive strategy for treatment in various disease areas. In this therapeutic approach, the CD3 complex is one of the key molecules to modulate T cell functions; however, in many cases, we cannot evaluate the drug candidates in animal experiments because the therapeutics, usually monoclonal antibodies specific to human CD3, cannot react to mouse endogenous Cd3. Although immunodeficient mice transfused with human hematopoietic stem or precursor cells, known as humanized mice, are available for these studies, mice humanized in this manner are not completely immune competent. In this study we have succeeded in establishing a novel mouse strain in which all the three components of the Cd3 complex — Cd3ε, Cd3δ, and Cd3γ — are replaced by their human counterparts, CD3E, CD3D, and CD3G. Basic immunological assessments have confirmed that this strain of human *CD3 EDG*–replaced mice are entirely immune competent, and we have also demonstrated that a bispecific antibody that simultaneously binds to human CD3 and a tumor-associated antigen (e.g. ERBB2 or GPC3) can be evaluated in human *CD3 EDG*–replaced mice engrafted with tumors. Our mouse model provides a novel means to evaluate the *in vivo* efficacy of human CD3–mediated therapy.

T cells are a type of lymphocytes that play a central role in adaptive immunity along with B cells. There are various types of T cells, such as helper, cytotoxic, regulatory T cells, etc., each of which has distinct characteristics and functions in the overall immune system^1^,^2^,^3^. The CD3 complex, a common surface marker on T cells, has important functions not only as an essential component in forming the T cell receptor (TCR)-CD3 complex, but also as an external signal transducer; therefore, the CD3 complex is one of the target molecules to modulate T cell functions. The first antibody to human CD3 to be approved was OKT3, which was developed to prevent rejection after organ transplantation^4^,^5^. Teplizumab and otelixizumab were then developed as second generation antibodies to CD3 to treat autoimmune type I diabetes^5^,^6^,^7^. More recently, novel therapeutic approaches have been developed for antitumor treatment using bispecific antibodies for human CD3 and a tumor-associated antigen (TAA) to simultaneously activate effector T cells and redirect them to the tumor cells. A large number of these bispecific antibodies that target human CD3 are already moving into the clinical phase — indeed, some are already approved — and the number is expected to increase in the near future^8^,^9^,^10^.

Most therapeutics that are being developed to target CD3 are molecularly targeted drugs, such as monoclonal antibodies that are highly specific to human CD3. It is therefore difficult to evaluate such therapeutics in preclinical examinations with animal models, because interspecies sequence preservation is relatively low in the extracellular domains of CD3ε (47% homology between human and mouse at the amino acid level, CD3δ (57% homology between human and mouse), or CD3γ (60% homology between human and mouse) (Supplementary Table S1 lists accession numbers and URL addresses for each protein). Although the laboratory mouse is an excellent experimental animal, therapeutics specific to human CD3 cannot effectively activate mouse effector T cells via their endogenous CD3 complex. Accordingly, an experimental animal model suitable for evaluating human CD3–specific therapeutics needs to be developed. In general there are two possible approaches to humanize CD3 in mice.

One approach is humanization by recapitulating the human hematopoietic system in immune-deficient mice^11^,^12^,^13^. These mice have a donor-derived human immune system that includes effector T cells. However, it is well known that several types of immune cells cannot develop and maturate normally in these mice^12^,^13^,^14^. Even though maturation of innate immune cells can be substantially improved by humanizing several cytokine genes, as Rongvaux *et al*. reported^15^, their adaptive immune function cannot be fully recovered, mainly because the major histocompatibility complexes (MHC) of the host cells and the human donor T cells are mismatched, which severely hampers the educational process and the peripheral survival of human T cells^16^. Suzuki *et al*. reported that a transgenic human MHC molecule, HLA-DR, taken from donors with compatible HLA type was effective for recapitulating the human adaptive immune system^17^. However, preparing a wide variety of HLA transgenic mice compatible with each donor HLA type would be a laborious challenge in large scale experiments. Another approach is to establish transgenic mice expressing the human CD3 complex. Of the three components in the CD3 complex, CD3ε is known to play the prerequisite role in formation and function of the TCR-CD3 complex, because *Cd3e*-knockout mice completely lack peripheral T cells, due to the blockade in early thymocyte development^18^,^19^,^20^. Human *CD3ε (CD3E*) transgenic mouse strains have already been reported^21^, but the number of thymocytes in these human *CD3E* transgenic mice is severely reduced. The degree of thymocyte depletion correlated with transgene copy numbers, and a higher transgene copy number resulted in complete loss of T cells^21^. Therefore, these human *CD3E* transgenic mouse strains would not be appropriate models to evaluate CD3-mediated therapeutics. From this evidence we speculated that the expression level of transgenic *CD3E* in human *CD3E* single transgenic mice would have to be precisely controlled and, furthermore, even if human CD3E expression could be appropriately controlled, its coexistence with endogenous Cd3e could affect the normal formation of TCR-CD3 complexes, because a highly complicated combination of the CD3 components would form on the T cells (as depicted in Supplementary Fig. S1), and this unnatural combination may result in relatively fewer T cells. Moreover, we hypothesized that affinity or compatibility of CD3E with the other two components, CD3 delta (CD3D) and CD3 gamma (CD3G), would be critical to form a normal CD3 complex.

In this study we have successfully established a novel mouse strain in which the entire CD3 components, i.e. CD3E, CD3D, and CD3G (referred to as CD3 EDG in this paper) were genetically humanized. This mouse strain has shown normal T cell development and maturation. Several immunological assessments *in vitro* and *in vivo* have proved that their immune functions, including the T cell functions, are normal. We expect that our mouse strain will contribute to developing human CD3–mediated therapeutics.

## Results

### Establishment of mice with entirely humanized CD3E, CD3D, and CD3G

**Figure 1:**
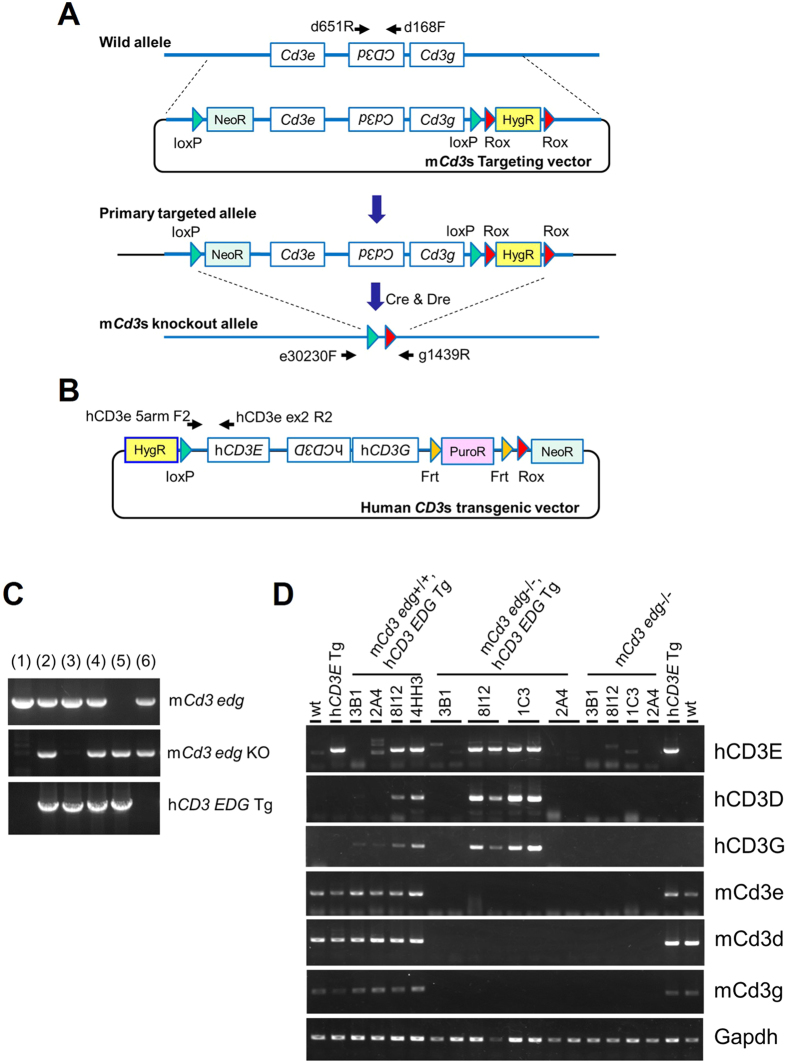
Schematic representation of the humanization strategy for *CD3E, CD3D*, and *CD3G*. (**A**) A vector targeting *Cd3 edg* was constructed by inserting loxP and Rox with *neo* and *hyg* cassette on a BAC genomic clone. Correctly targeted ES cell clones with the *Cd3 edg*–targeting vector had the primary targeted allele. Next, mouse *Cd3 edg* genomic region flanked by two loxP sequences and the *hyg* cassette flanked by two Rox sequences were deleted by Cre and Dre recombinase. Arrows, PCR primers for genotyping. (**B**) Human *CD3 EDG* transgenic vector was introduced to primary targeted ES cell clones at the same time as the *Cd3 edg* deletion described above. Arrows, PCR primers for genotyping. (**C**) A representative result of genotyping to confirm deletion of endogenous *Cd3 edg* locus and integration of the human *CD3 EDG* transgene. Information of the primer set to detect each allele is listed in Supplementary Table S3. Wild allele and the disrupted allele for mouse *Cd3 edg* were detected as signals of 0.8 kb and 1.4 kb, respectively. The human *CD3 EDG* transgene was detected as a signal of 5.5 kb. Numbers above each gel denote the mouse genotypes, (1) wild-type (m*Cd3 edg*+/+), (2) m*Cd3 edg*+/−, h*CD3 EDG* Tg, (3) m*Cd3 edg*+/+, h*CD3 EDG* Tg, (4) m*Cd3 edg*+/−, h*CD3 EDG* Tg, (5) m*Cd3 edg*−/−, h*CD3 EDG* Tg (human *CD3 EDG*–replaced), and (6) m*Cd3 edg*+/−. This figure combines images cropped from the original gel electrophoresis images, which are shown in Supplementary Fig. S2A and B. (**D**) Representative results of RT-PCR analysis for mouse *Cd3 edg* or human *CD3 EDG* expression in the blood cells. This figure combines images cropped from the original gel electrophoresis images, which are shown in Supplementary Fig. S3A–E.

The vector construction and recombination strategy used to establish entirely humanized *CD3 EDG*–replaced mice are depicted in Fig. 1A and B. Several clones of C57BL/6N (B6) mouse embryonic stem (ES) cells (clones 1C3, 2A4, 3B1, 4HH3, and 8I12) that had been successfully recombined after two rounds of recombination were microinjected to BALB/cA mouse blastocysts to make chimeric mice. Male chimeric mice were bred with female B6 mice to obtain their offspring with a knockout allele for the entire mouse *Cd3 edg* and a human *CD3 EDG* transgenic allele. After breeding the offspring from these five ES clones, five strains of endogenous C*d3 edg* homozygous knockout with entire human *CD3 EDG* transgenic mice (referred to as human *CD3 EDG*–replaced mice), lines 1C3, 2A4, 3B1, 4HH3, and 8I12, were established. Representative results of genotyping PCR are shown in Fig. 1C. Neither Cre nor Dre vectors were integrated in the mouse genome. Human *CD3 EDG*–replaced mice were obtained in accordance with Mendelian laws. No apparent gross abnormalities were observed in any strains of human *CD3 EDG*–replaced mice. The *Cd3 edg*–knockout mice (referred to as *Cd3 edg*^−/−^ mice) obtained in this breeding process have no apparent gross abnormalities other than in the immune system. These mouse lines were listed in Supplementary Table S2.

### Establishment of humanized CD3E transgenic mice

As we predicted from the previous publication by Wang *et al*.^21^, it was difficult to obtain a transgenic mouse with an appropriate human *CD3E* expression level. In total, seventeen founders were obtained by pronuclear DNA microinjection or electroporation to mouse ES cells of the human *CD3E* transgenic constructs. The human *CD3E* transgene was transmitted from all founders to their offspring to establish 17 lines of transgenic mice. Of these lines, one line of the transgenic mice exhibited a desirable response in an *in vitro* cytotoxicity assay (data not shown), in which splenocytes from transgenic mice were incubated with a cell line expressing the TAA and a bispecific antibody to both human CD3 and the TAA. This line of transgenic mice (referred to as h*CD3E* Tg mice in this paper) was compared with human *CD3 EDG*–replaced mice for further investigation. The characteristics of the transgenic mouse lines established in this study have been listed in Supplementary Table S2.

### Transgene expression of human CD3 components in the human CD3 EDG–replaced mice

Figure 1D shows representative results of the reverse transcription (RT) PCR to assess mouse *Cd3 edg* and transgenic human *CD3 EDG* expression in blood cells of entirely humanized *CD3 EDG* mice. In these results, endogenous mouse *Cd3 edg* was not detected in any of the lines tested. Transgenic human *CD3 EDG* expression was detected in three lines, (1C3, 4HH3, and 8I12) and thus these three lines were used for further analysis, described below. On the contrary any of the transgenic human *CD3 EDG* expression was not detected in remaining two lines of mice, 2A4 and 3B1.

**Figure 2:**
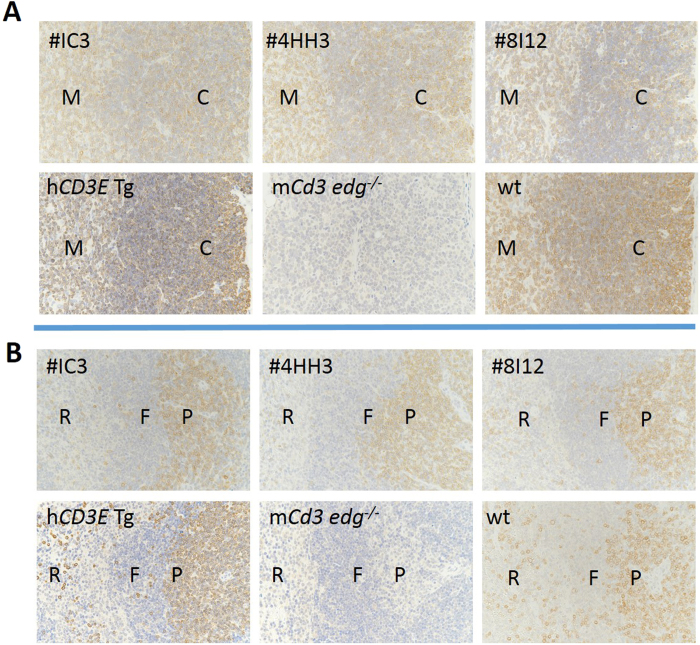
Immunohistochemical staining for CD3 in (**A**) the thymus and (**B**) the spleen. The T cell zone in each tissue was specifically stained for CD3 in human *CD3 EDG*–replaced mice and in wild-type mice. No staining was detected in the tissues of *Cd3 edg*−/− mice. wt, wild-type mice; m*Cd3 edg*−/−, mouse *Cd3 edg* knockout mice; h*CD3E* Tg, human *CD3E* transgenic mice; 4HH3, 8I12 and 1C3 indicate the three different strains of human *CD3 EDG*–replaced mice; M, medulla; C, cortex; P, periarteriolar lymphoid sheaths (PALS); F, follicles; R, red pulps; In m*Cd3 edg*^−/−^, the boundary between cortex (C) and medulla (M) was not clear in either the thymus or the spleen.

The results of immunohistochemical staining show that CD3-positive (CD3+) cells were located specifically in the T cell zones in the thymus and spleen of human *CD3 EDG*–replaced mice, just as they are in wild-type mice, whereas no staining was observed in any tissues from the *Cd3 edg*^−/−^ mice (Fig. 2). The CD3+ cells were morphologically normal lymphocytes. A small number of CD3+ cells sporadically distributed in all the organs of human *CD3 EDG*–replaced mice were morphologically normal lymphocytes, as were those of wild-type mice (Table 1 and Supplementary Fig. S4). No ectopic CD3 expression was observed in any tissues of the human *CD3 EDG*–replaced mice, other than in lymphocytes, as far as we examined.

### Basic characteristics of the human CD3 EDG–replaced mice

**Figure 3:**
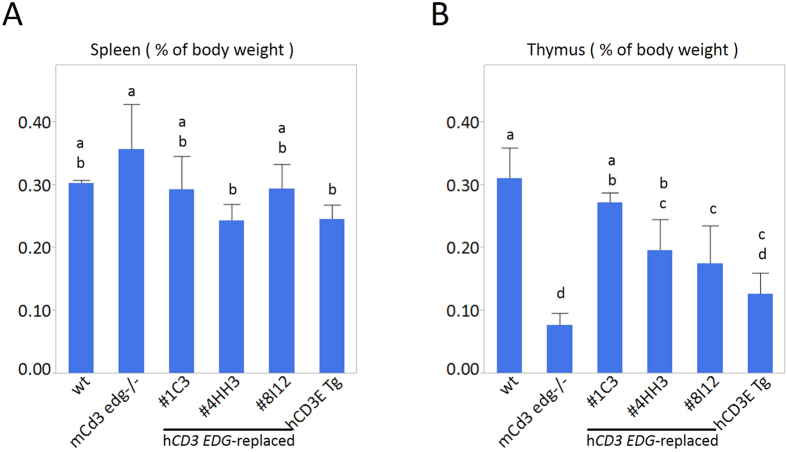
Weight of (**A**) spleens and (**B**) thymuses. Organ weight is shown as a ratio to body weight (n = 4, each genotype). Data are represented as mean ± s.d. In bars marked with different letters (a b, c, and d) the data sets are significantly different by Tukey-Kramer’s HSD tests, P < 0.05. Detailed data on *p* values between all combinations of genotypes are shown in Supplementary Tables S5A and B. wt, wild-type; m*Cd3 edg*−/−, mouse *Cd3 edg* knockout mouse; 1C3, 4HH3, and 8I12 indicate the strain names of human *CD3 EDG*–replaced mice; h*CD3E* Tg, human *CD3E* transgenic mouse.

The three lines of human *CD3 EDG*–replaced mice (1C3, 4HH3, and 8I12), the *Cd3 edg*^−/−^ mice, and the h*CD3E* Tg mice were not significantly different in body weight (data not shown) or spleen weight (Fig. 3A) compared to wild-type mice. No anatomical abnormalities were observed in their tissues except in the thymus (data not shown). Both *Cd3 edg*^−/−^ mice and h*CD3E* Tg mice have apparently atrophic thymuses. Averages of thymus weights in *Cd3 edg*^−/−^ mice and h*CD3E* Tg mice were decreased by 25% and 41% that of wild-type mice, respectively. Averages of thymus weights in the three human *CD3 EDG–*replaced mouse lines (1C3, 4HH3, and 8I12) appeared to be, respectively, 88%, 63%, and 57% that of the wild-type mice (Fig. 3B). Of the three lines of human *CD3 EDG*–replaced mice, thymus weights in one line (1C3) were similar to those in wild-type mice (Fig. 3B) and thymus weights in the other two lines (8I12 and 4HH3) were significantly lower than those in wild-type mice. Although thymus sizes varied between the transgenic lines, all three lines (1C3, 4HH3, and 8I12) of human *CD3 EDG*–replaced mice had morphologically normal thymuses (data not shown). Total cell numbers either in the spleens or the thymuses of human *CD3 EDG*–replaced mouse line 1C3 were not significantly different from those in wild-type mice (Supplementary Fig. S5).

### Splenocyte characteristics in the human CD3 EDG–replaced mice

**Figure 4:**
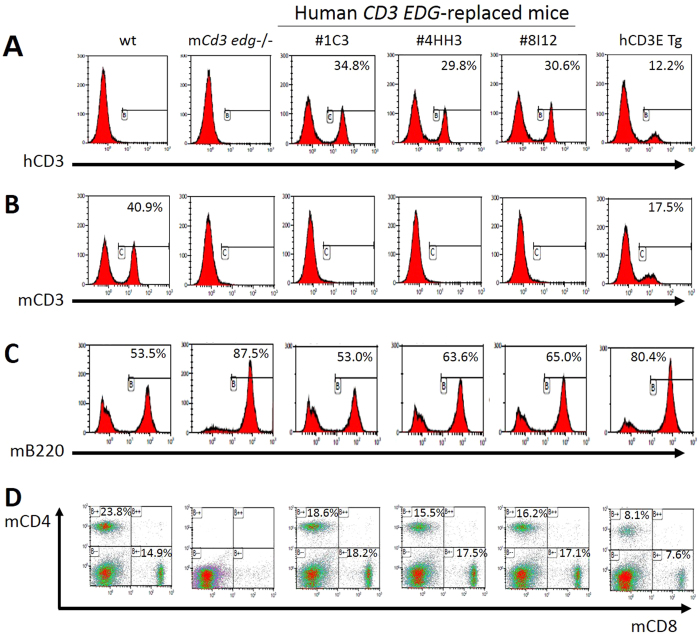
Representative results of a flow cytometric analysis of human *CD3 EDG*–replaced mice, *Cd3 edg*^−/−^, and wild-type mice. Distribution of populations of (**A**) human CD3, (**B**) mouse CD3, (**C**) B220, and (**D**) mouse CD4- or CD8-single positive cells were analyzed using splenocytes prepared from each mouse genotype. Numbers in the histogram or quadrants indicate percentage within the gated cells. wt, wild-type mice; m*Cd3 edg*−/−, mouse *Cd3 edg* knockout mice; 1C3, 4HH3 and 8I12 indicate the strains of human *CD3 EDG*–replaced mice; h*CD3E* Tg, human *CD3E* transgenic mouse. All studies were representative of two to three experiments.

We analyzed lineage marker expression of T and B cells in the splenocytes of human *CD3 EDG*–replaced mice, and compared the expression profiles with those in h*CD3E* Tg mice and wild-type mice. Flow cytometric analysis demonstrated that human CD3+ cells were detected in splenocytes in three lines of human *CD3 EDG*–replaced mice (1C3, 8I12, and 4HH3) (Fig. 4A). Certainly, mouse Cd3+ cells were not detected in these mice (Fig. 4B). Of these three lines, the line 1C3 showed the highest expression of human CD3, similar to the level of mouse Cd3+ cells in wild-type mice (Fig. 4A and B). These results clearly demonstrated that mouse Cd3 was successfully replaced with human CD3 in our human *CD3 EDG*–replaced mice. By contrast, human CD3+ cell populations in h*CD3E* Tg mice were much lower than those in human *CD3 EDG*–replaced mice. Mouse Cd3+ cell populations were lower in h*CD3E* Tg mice than those in wild-type mice, even though h*CD3E* Tg mice have an intact endogenous *Cd3 edg* genomic locus. These results are compatible with the description by Wang *et al*.^21^. Populations of B220+ cells, which are mainly expressed by the B lymphocytes, in all three lines of human *CD3 EDG*–replaced mice were similar to those in wild-type mice (Fig. 4C), whereas B220+ cells in *Cd3 edg*^−/−^ mice and in h*CD3E* Tg mice were greater in number than those in wild-type mice. The cause of these increased populations of B220+ cells remains to be investigated, but we speculated that there might be a redundant mechanism that maintains the spleen tissue structure in response to T cell depletion or reduction.

In all three lines of human *CD3 EDG*–replaced mice, single positive cell populations of CD4 T cells or CD8 T cells were detected (Fig. 4D). Of the three lines, the matured T cell populations in line 1C3 were most similar to those in wild-type mice. Human CD3 derived from transgene was co-expressed with TCR beta in the 1C3 line of human *CD3 EDG*–replaced mice (Supplementary Fig. S6A). The percentage of matured T cell populations, i.e. CD3+CD4+ and CD3+CD8+ cells, in the 1C3 line were similar to those in wild-type mice (Supplementary Fig. S6B and C). On the other hand, neither CD4+ nor CD8+ cells were detected in *Cd3 edg*^−/−^ mice, and populations of CD4+ or CD8+ cells in h*CD3E* Tg mice were decreased to about half the levels seen in wild-type mice. According to the results above, we selected the 1C3 line of human *CD3 EDG*–replaced mice.

### Response of splenocytes to mitogenic stimulation

**Figure 5:**
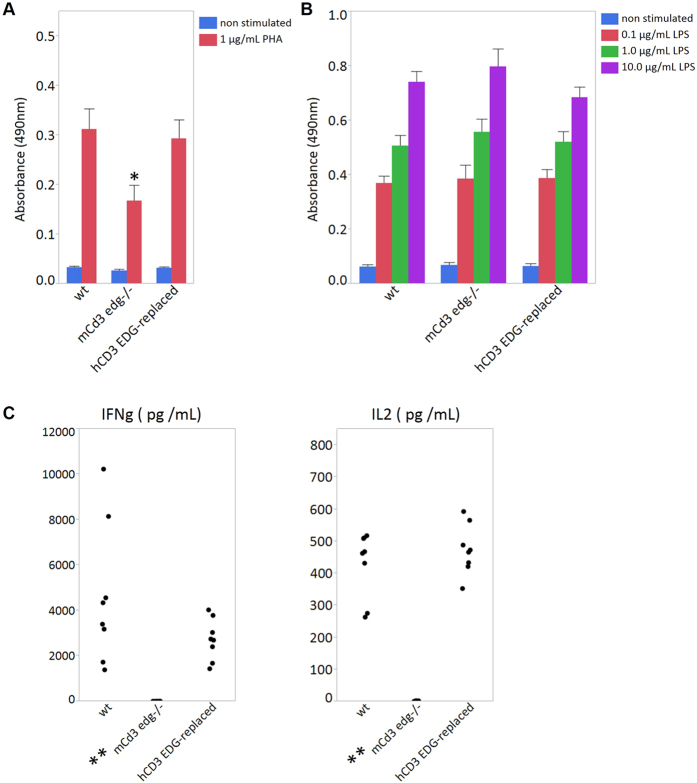
Proliferative response to mitogen stimulation. Splenocytes were cultured (**A**) for 3 days stimulated with 1.0 μg/mL of PHA (red bar) or without stimulation (blue bar) and (**B**) for 2 days stimulated with 0.1 (red bar), 1.0 (green bar), or 10.0 μg/mL (purple bar) of LPS or without stimulation (blue bar). Splenocyte proliferation was analyzed by MTS assay (Promega), and the results indicated the mean absorbance at 490 nm. Data are represented as mean value ± s.e.m (n = 8). (**C**) Evaluation of the cytokine production with PHA stimulation. Splenocyte cells were stimulated with 1.0 μg/mL of PHA and the culture supernatants were harvested 3 days after stimulation. Cytokine levels (pg/mL) of IFN gamma and IL2 in culture medium were measured using Cytometric Bead Array (BD Biosciences). Data is significantly different compared to wild type mice. (*P < 0.05, **P < 0.0001 by Dunnett’s test for multiple comparisons.) Data are shown as dot plots (n = 8). wt, wild-type mice; m*Cd3 edg*−/−, mouse *Cd3 edg* knockout mice; hCD3 EDG-replaced, human *CD3 EDG*–replaced mice (1C3).

According to the results of flow cytometric analysis, not only T cell depletion but also abnormally increased populations of B cells were observed in the spleen of *Cd3 edg*^−/−^ mice (Fig. 4C). Thus, we needed to confirm that humanization of *CD3 EDG* would not affect development and proliferation of B cells. Therefore, we examined the proliferative activity of splenocytes in response to not only T cell mitogen but also B cell mitogen using phytohaemagglutinin (PHA) as a T cell mitogen (Fig. 5A) and lipopolysaccharide (LPS) as a B cell mitogen (Fig. 5B). There was no significant difference in responses to PHA between wild-type mice and human *CD3 EDG*–replaced mice, whereas proliferative activity was significantly reduced in *Cd3 edg*^−/−^ mice. Although the response observed in *Cd3 edg*^−/−^ mice was higher than that in the unstimulated control, we suppose that this would be due to the response by immune cells other than mature T cells in the spleen, because PHA does not exclusively stimulate T cells, but also B cells, as previously reported^22^. As for LPS, substantially similar dose-dependent proliferative responses were observed in human *CD3 EDG*–replaced mice, *Cd3 edg*^−/−^ mice, and wild-type mice.

To evaluate cytokine production related to T helper cells, the splenocytes were stimulated with PHA, and the cytokine concentrations in the culture supernatants were assessed (Fig. 5C). As a result, IFN gamma and IL2 in human *CD3 EDG*–replaced mice were detected at similar levels to those in wild-type mice. In contrast, these cytokines were produced very slightly in *Cd3 edg*^−/−^ mice. These results indicate that human *CD3 EDG*–replaced mice have immunological competency in mitogenic stimulations.

**Figure 6:**
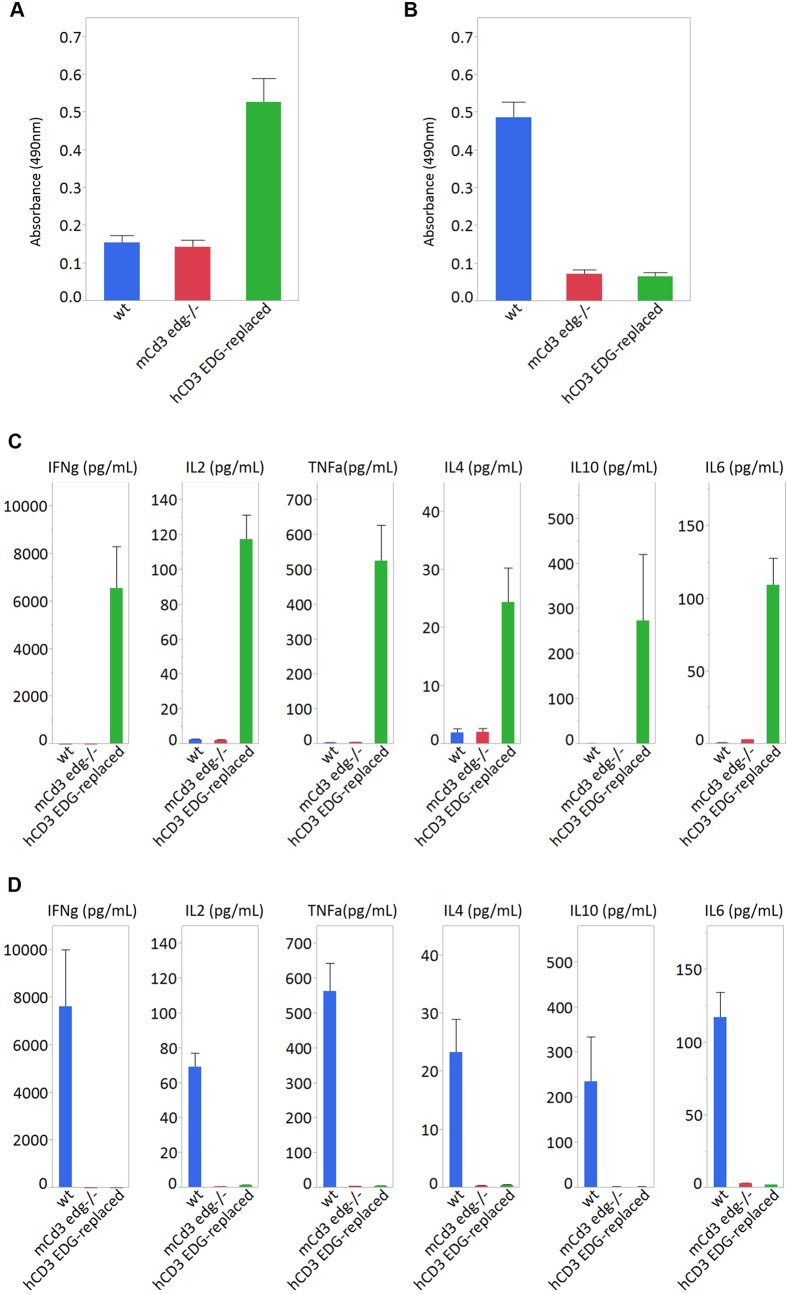
Representative results of an MTS assay to evaluate cell proliferation after stimulation with an antibody specific to mouse or human CD3. Splenocytes were stimulated with anti-human CD3 antibody (**A**,**C**) and anti-mouse CD3 antibody (**B**,**D**). After 3 days stimulation, splenocyte proliferation was analyzed by MTS assay (Promega) (**A**,**B**). Cytokine productions were determined in human *CD3 EDG*–replaced mice, *Cd3 edg*^−/−^ mice, and wild-type mice (**C**,**D**). The culture supernatants were harvested 3 days after stimulation and cytokine levels (pg/mL) of IFN gamma, TNF alpha, IL6, IL10, IL4, and IL2 in culture medium were measured using Cytometric Bead Array (BD Bioscience). Data are represented as mean value ± SEM (n = 8). wt, wild-type mice; m*Cd3 edg*−/−, mouse *Cd3 edg* knockout mice; hCD3 EDG-replaced, human *CD3 EDG*–replaced mouse line 1C3.

Next, we examined whether T cells in human *CD3 EDG*–replaced mice would respond to stimulation with agonistic antibodies specific to human CD3 and mouse Cd3, and compared the results with the responses to agonistic antibodies specific to mouse Cd3 and human CD3 in wild-type mice. As a result, splenocytes from human *CD3 EDG*–replaced mice could proliferate (Fig. 6A) and produce cytokines in response to the human CD3 antibody (Fig. 6C), but did not respond to the mouse Cd3 antibody. Meanwhile, splenocytes from wild-type mice could respond to the mouse Cd3 antibody (Fig. 6B and D), but could not respond to the human CD3 antibody.

### Specific antibody production in response to immunization with an external antigen, ovalbumin (OVA)

**Figure 7:**
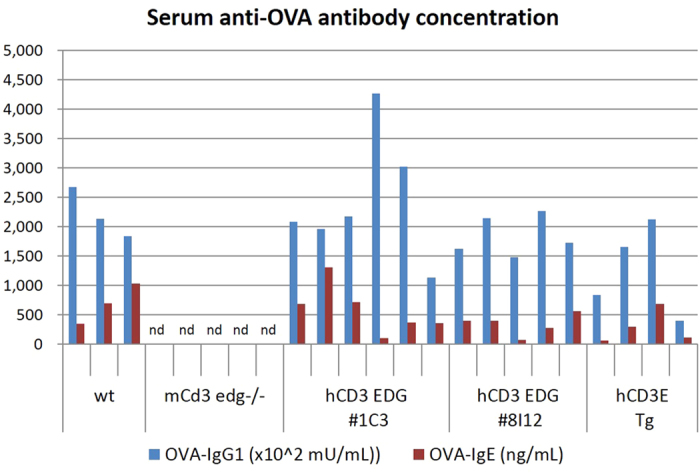
Serum titers of OVA-specific IgE- and IgG1-class antibodies. Mice were immunized with OVA twice, 4 weeks apart. Blood samples were collected a week after the second immunization. Each bar represents serum concentration of OVA specific-IgG1 or IgE of individual mice. wt, wild-type mice; m*Cd3 edg*−/−, mouse *Cd3 edg* knockout mice; hCD3 EDG, human *CD3 EDG*–replaced mice; #1C3 and #8I12 indicate strains of human *CD3 EDG*–replaced mice; h*CD3E* Tg, human CD3E transgenic mouse.

After the second immunization of OVA with Freund adjuvant, both the OVA-specific immunoglobulin (Ig) E- and Ig G1-class antibodies were detected in the serum of human *CD3 EDG*–replaced mice (Fig. 7). The levels of OVA-specific IgE and IgG1 titers were similar to those in wild-type mice. In contrast, neither OVA-specific IgE nor IgG1 titers were detected in the serum of *Cd3 edg*^−/−^ mice. These results indicate that human *CD3 EDG*–replaced mice respond to immunization with external antigens by producing normal levels of antibody.

### T cell activation with hCD3-TAA bispecific antibodies in human CD3 EDG–replaced mice

**Figure 8:**
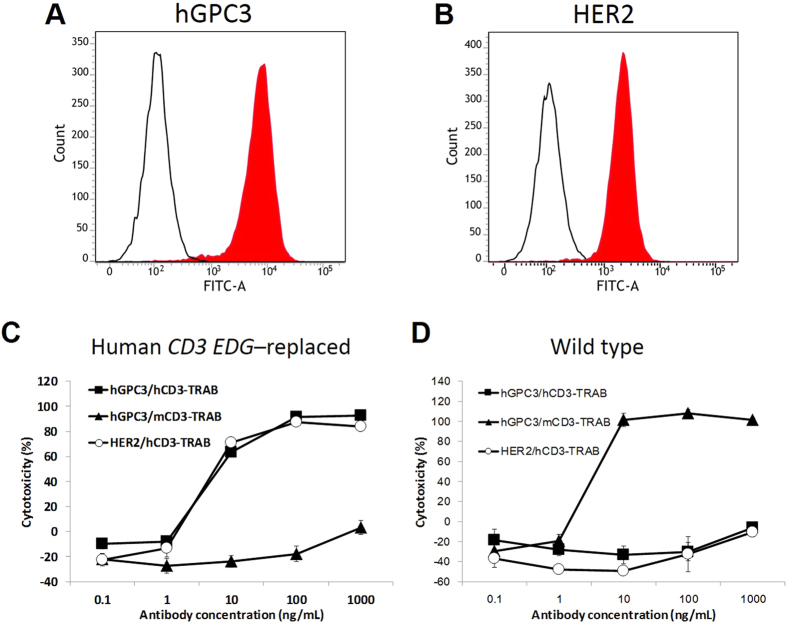
TRAB-mediated killing by splenic T cells of human *CD3 EDG*–replaced mice. Surface expression of (**A**) GPC3 and (**B**) HER2 was determined on Hepa1-6/hGPC3/HER2 cells by FACS. T cells were extracted from the spleens of (**C**) human *CD3 EDG*–replaced mice or (**D**) C57BL/6 mice. *In vitro* killing activity of Hepa1-6/GPC3/HER2 cells was tested using human CD3 TRABs specific to hGPC3 or HER2, and a mouse CD3 TRAB specific to hGPC3. E:T = 40:1 Assay time: 72 hours. *In vitro* cytotoxicity was monitored by xCELLigence.

**Figure 9:**
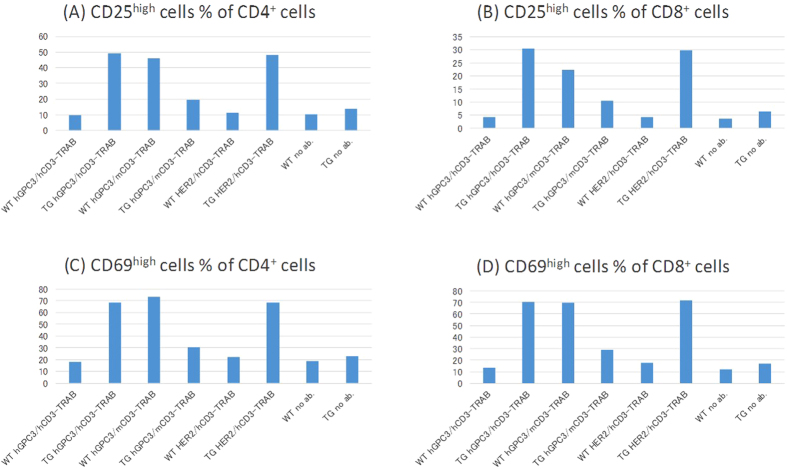
Target-dependent T cell–activation. The expression levels of activation markers CD25, CD69, on (**A**,**C**) CD4+ or (**B**,**D**) CD8+ T cells were determined by flow cytometry. T cells were prepared from the *in vitro* tumor cell killing assay in Fig. 8, at the 72 hours timepoint. WT, wild type mouse; TG, human *CD3 EDG*–replaced mouse; no ab, without antibody administration.

We tested T cell–dependent cellular cytotoxicity (TDCC) using a T cell–redirecting antibody (TRAB). In this TDCC assay, the effector cells were spleen cells of human *CD3 EDG*–replaced mice and the target cells were Hepa1-6/hGPC3/HER2 cells, in which human glypican-3 (GPC3) and erb-b2 receptor tyrosine kinase 2 (HER2) are significantly expressed in Hepa1-6 mouse hepatocellular carcinoma (Fig. 8A and B). TRABs bispecific to hCD3 and either HER2 or hGPC3 showed TDCC (described as cytotoxicity (%) calculated by equation (1) in the Methods section) with effector cells from human *CD3 EDG*–replaced mice (Fig. 8C), but did not show TDCC with effector cells from wild-type mice (Fig. 8D). On the other hand, a TRAB bispecific to mCD3 and hGPC3 showed TDCC with effector cells from wild-type mice, but did not show TDCC with effector cells from human *CD3 EDG*–replaced mice. These results demonstrated that TDCC induced by TRABs is dependent on the combinations of antibody with target CD3 and on the genotype of effector cells. To confirm the activation of T cells, we then examined the expression levels of CD69 (an early marker of T cell activation), CD25 (a late marker of T cell activation), CTLA-4 (CD152), and ICOS (CD278). As shown in Fig. 9A–D, CD25 and CD69 expression increased on T cells derived from human *CD3 EDG*–replaced mice treated with TRABs bispecific to hCD3 and either HER2 or hGPC3, whereas when a TRAB bispecific to mCD3 and hGPC3 was used, CD25 and CD69 expression was increased on T cells derived from wild type mice, but not on T cells derived from human *CD3 EDG*–replaced mice. Changes in CTLA-4 and ICOS expression were not observed clearly in these samples (data not shown).

**Figure 10:**
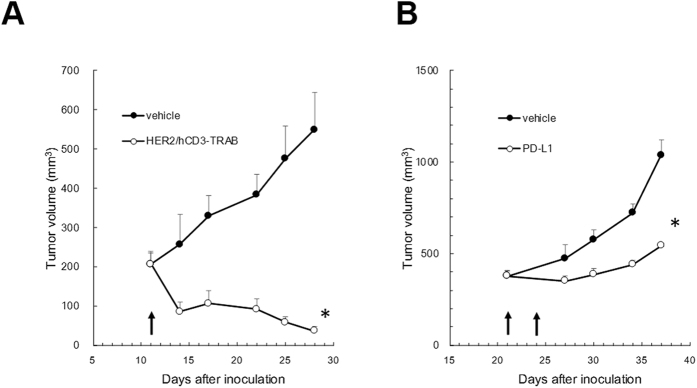
A TRAB bispecifically targeting hCD3 and HER2 or anti-PD-L1 antibody inhibits *in vivo* growth of tumor in the human *CD3 EDG*–replaced mouse model. A total of 1 × 10^7^ Hepa1-6/hGPC3/HER2 cells or Hepa1-6/hGPC3 were inoculated together with 50% matrigel-matrix (BD Bioscience). (**A**) Mice were treated with 5 mg/kg i.v. doses of the TRAB (n = 4) or the vehicle (n = 5) on Day 11 after tumor inoculation. (**B**) Mice (n = 5) were injected with a 10-mg/kg i.v. dose of anti-PD-L1 antibody on Days 21 and 24. Arrows show the time of antibody injection. HER2/hCD3-TRAB: bispecific antibody to hCD3 and HER2 (*P < 0.05 by Wilcoxon test).

When the antitumor effect of the TRAB to HER2 was examined in a human *CD3 EDG*–replaced mouse model engrafted with Hepa1-6/hGPC3/HER2 cells, the antitumor effect was significant, with 149% tumor growth inhibition (TGI, calculated by equation (2) in the Methods section) at 28 days after tumor implantation (Fig. 10A). The antitumor effect of the PD-L1 antibody was also observed in a human *CD3 EDG*–replaced mouse model engrafted with Hepa1-6/hGPC3 cells. The antitumor effect was significant, with 75% TGI at 37 days after tumor implantation (Fig. 10B).

## Discussion

This report explains how we successfully established a novel mouse strain that has an entirely humanized CD3 complex by disrupting the endogenous genome covering *Cd3e, Cd3d*, and *Cd3g* and simultaneously introducing a complete genomic region of human *CD3E, CD3D*, and *CD3G*. The most important advantage of our human *CD3 EDG*–replaced mouse strain is in immune competence. Our mouse strain is immunologically normal in tests so far. First, neither T cell reduction nor thymus hypoplasia were observed in the human *CD3 EDG*–replaced mouse line 1C3 (Fig. 3B and Supplementary Fig. S5), whereas both abnormalities were previously reported in h*CD3E* Tg mice and also reconfirmed in this study (Fig. 3B). The thymus weights observed in the human *CD3 EDG*–replaced mice varied between the transgenic lines. We speculate that there may be some subtle differences in transgene expression levels between the three lines, possibly due to the effect of some neighboring genomic elements, e.g. enhancers, suppressors, genomic structure, etc., that surround the transgene integration site of each line. Secondly, T cell differentiation into mature CD4 or CD8 single positive T cells is normal in the human *CD3 EDG*–replaced mice (Fig. 4D and Supplementary Fig. S6B and C). It is well known that appropriate signals from TCRs at an appropriate time are essential for normal T cell development and differentiation in the thymus^23^. According to an MTS assay, the T cell proliferative response is normal after several mitogen stimulations on the spleen cells (Fig. 5A and C). A species-specific agonistic antibody to CD3 can differentially stimulate splenocytes according to their genotypes (Fig. 6A–D), which demonstrates that not only has endogenous mouse Cd3 edg been replaced with human CD3 EDG but also human CD3 EDG functions normally on T cells in mice. Thirdly, helper T cells in our mice stimulate B cells normally to produce Ig E and Ig G1 antibodies specific to a foreign antigen, OVA (Fig. 7), indicating that Ig isotype switching is also taking place normally. It is well known that CD4^+^ helper T cell functions are essential, not only to produce specific antibodies but also for antibody isotype switching and affinity maturation^24^,^25^. These results demonstrated that CD4^+^ helper T cells function normally in our human *CD3 EDG*–replaced mice. Although the function of regulatory T cells remains to be investigated, it is expected that they would develop and function normally because human *CD3 EDG*–replaced mice do not show signs of autoimmunity^3^. The results above clearly show that our human *CD3 EDG*–replaced mice are immunologically competent. We expect that our human *CD3 EDG*–replaced mice will contribute especially to the development of therapeutics, either antibodies for use in the extracellular domain or small molecule drugs etc. for use in the cytoplasmic domain, to treat cancer or the autoimmune diseases.

Recent advances in antibody-engineering technology make it possible to develop not only target-specific therapeutics but also antibodies with various functions, such as bispecific target-binding, agonistic activity, conditional target-binding properties, and effector cell redirection^8^,^26^,^27^,^28^,^29^. Of these antibody-based therapeutics, TRABs are attracting attention in the field of cancer immunotherapy^8^. A TRAB is a type of bispecific antibody that can simultaneously bind to a target antigen on the tumor cell and to CD3 to activate T cells residing in the tumor through the CD3 complex, without interacting with antigen-presenting cells and thus independent of their TCR-binding specificity. Antibody engineering has been used to develop numerous biologics of this type, including the bispecific T Cell Engager (BiTE) antibodies^9^. For clinical trials of these therapeutic antibodies to be successful, their efficacy needs to be adequately evaluated in preclinical experiments; however, as noted earlier, interspecies sequence preservation is relatively low in the extracellular domains of either CD3ε, CD3δ, or CD3γ. In many cases, humanized mouse models, which are immune-deficient mice transfused with human hematopoietic stem or precursor cells, have been widely used for preclinical evaluation of therapeutics that specifically target human molecule(s) on blood cells, such as immune cells^11^,^12^,^13^,^14^, but these humanized mouse models are not completely immune-competent either in terms of innate immunity or adaptive immunity. To resolve the lack of immune-competent animal models for evaluating antibody-based therapeutics that target human CD3, we examined whether our novel human *CD3 EDG–*replaced mouse model can be used to evaluate the *in vivo* efficacy of TRABs. As a result, complete growth inhibition was observed after administering a TRAB against HER2 and human CD3 in a model engrafted with syngeneic tumor cells that express both human HER2 and human GPC3 (Fig. 10A), and several *in vitro* experiments demonstrated that the growth inhibition effect was via TDCC activity (Figs 8 and 9). Moreover, the study showed that inhibiting the immune checkpoint pathway through PD1 and PDL1 can result in growth inhibition of grafted tumor cells (Fig. 10B), which indicates that effector T cells in human *CD3 EDG–*replaced mice can be normally activated by immune checkpoint inhibitors.

In any case, only using conventional immunodeficient mouse models would not be sufficient to precisely evaluate drug candidates. We would like to propose the importance of evaluating drug candidates in multiple humanized animal models: one is the conventional model that recapitulates the human immune system in immunodeficient mice, and another is an immune-competent mouse model like our human *CD3 EDG–*replaced mouse. Nowadays, numerous novel therapeutics are being developed for cancer immunotherapy and autoimmune diseases using the latest advances in drug-engineering technologies. We hope that our human *CD3 EDG–*replaced mice can contribute to the development of these innovative drugs in the near future.

## Methods

### Generation of entire human CD3E, CD3D, and CD3G–replaced mice

Bacterial artificial chromosome (BAC) genomic DNA clones that cover the entire mouse *Cd3* gene region (Invitrogen Clones, RPCI23.C., Clone#410N16)^30^ or the human CD3 gene region were purchased from Life Technologies (Invitrogen Clones, RPCI11.C., Clone#30E1)^31^.

A targeting vector was constructed to disrupt the entire genomic region covering mouse Cd3 (*Cd3e, Cd3d*, and *Cd3g*). A BAC genomic DNA clone that covers the entire mouse *Cd3* gene region was used to construct the targeting vector by using Red/ET system (Gene Bridges, GmbH)^32^,^33^. At first, a loxP site with *neo* (loxP-*neo*) cassette was inserted at 5′ upstream of *Cd3e* on the genome in the BAC clone, and simultaneously 5′ upstream region was shaved to approximately 3.5 kb as a 5′ homology arm. Next, another loxP site and a hygromycin resistance gene (hygR) cassette flanked by two Rox sites (loxP-Rox-hygR-Rox cassette) were inserted at 3′ downstream of *Cd3g*. Finally 3′ downstream region of *Cd3g* was shaved to approximately 3.4 kb as a 3′ homology arm.

The vector introducing the entire human CD3 gene (*CD3E, CD3D*, and *CD3G*) was constructed by recombination of the genomic region cloned in the BAC vector with Red/ET system. The 5′ upstream genomic region of *CD3E* was shaved to 6.5 kb by simultaneous insertion of a hygromycin resistance cassette. Next, 3′ downstream region of *CD3G* was shaved to approximately 4.2 kb by simultaneous insertion of a dual drug selection marker, which consists of a puromycin resistance gene cassette flanked with Frt and a *neo* cassette with a Rox site (Fig. 1B).

Human *CD3 EDG*–replaced mouse ES cell lines were established by two rounds of recombination protocol as described below. At the first recombination, mouse *Cd3* targeting vector was electroporated to C57BL/6N (B6) mouse ES cells. The ES cells were selected in a culture medium containing G418. G418-resistant ES cell clones were screened by PCR protocol to confirm that both loxP-*neo* cassette and loxP-Rox-hygR-Rox cassette were correctly inserted in the target genomic sites. Correctly mutated ES cell clones (referred to as primary mutant ES cell clones) were expanded for the second recombination. The vector introducing the entire human *CD3 EDG* gene was electroporated to the primary mutant ES cell clones with Cre and Dre expression vectors. The ES cells were selected in a medium containing puromycin. Puromycin-resistant ES cell clones were screened by PCR protocol to confirm integration of the human CD3 genomic DNA and complete the deletion of mouse endogenous Cd3 genomic region. Correctly mutated ES cell clones were injected into BALB/cA mouse (CLEA Japan, Tokyo) blastocysts to produce chimeric mice. Chimeric mice were bred with B6 females to establish human *CD3 EDG*–replaced mouse lines. Genotyping was performed by genomic PCR in this breeding process. Primer sets, used for endogenous mouse *Cd3* locus, mouse *Cd3 edg*−/− locus and transgenic human *CD3 EDG*, are listed in Supplementary Table S3. After several assessments for transgene expression and basic immunological tests, we selected the 1C3 line of h*CD3 EDG*–replaced mice (officially named C57BL/6N-Del(9Cd3e-Cd3g)1Csk-Tg(CD3E, CD3D, CD3G)1Csk).

#### Generation of human CD3E transgenic mice

Human *CD3E* transgenic mice (official strain name: C57BL/6N-Tg(hCD3E)301Csk) were established by basically the same protocol as that described previously by Wang^21^, except that the vector was constructed with the Red/ET system. The genomic region, containing all the exons and introns of *CD3E* with 6.5 kb of 5′ upstream region and 8 kb downstream region, was separated by pulsed-field gel electrophoresis and microinjected into the pronucleus of B6 mouse–fertilized eggs.

#### RT-PCR for evaluation of transgenic human CD3 EDG and mouse endogenous Cd3 edg gene expression

RT-PCR analysis was performed to determine human *CD3 EDG* and mouse *Cd3 edg* gene expression. Total RNA samples, which were purified from mouse blood with Catrimox-14 RNA Isolation Kit (TaKaRa Bio, WA005, Japan) or NucleoSpin RNA Blood Kit (TaKaRa Bio, U0200B, Japan), were reverse-transcribed with SuperScript III reverse-transcriptase (Invitrogen) to synthesize cDNA. PCRs were performed with the gene-specific primers listed in Supplementary Table S4.

#### Immunohistochemical staining of CD3

Tissue samples were fixed in 10% neutralized formalin and were embedded in paraffin blocks. Antigen retrieval was performed for the tissue sections from these paraffin blocks by microwave oven for 10 min in Target Retrieval Solution (DAKO, S1699). After incubation in 30% hydrogen peroxide in methanol for 30 min to block endogenous enzyme activity, tissue sections were incubated in working solution of M.O.M.™ Mouse IgG Blocking Reagent (Vector^®^ M.O.M.™ Immunodetection Kit) for 1 hour and then in 5%BSA/TBS buffer for 30 min. Monoclonal anti-human CD3 (2.5 μg/mL, DAKO F7.2.38) was used as a primary antibody for incubation overnight at 4 °C. After incubation with secondary antibody, rat anti-mouse IgG heavy chain (LO-MG1-13: Abcam ab11585), biotin-labelled rabbit anti-rat IgG (5.0 μg/mL #A110-322, Bethyl), and HRP-labeled streptavidin (5.0 μg/mL, Vector SA-5740) were applied according to the standard ABC method. Tris-HCl buffer with 0.05% 3.3′-diaminobenzidine (DAB) in 0.03% hydrogen peroxide was used for staining.

#### Flow cytometry analysis

Primary splenocytes were isolated from each mouse by mechanical dissociation followed by red blood–cell lysis. These splenocytes were stained with the following monoclonal antibodies: FITC labeled anti-human CD3 (clone; UCHT1), FITC labeled anti-mouse CD3 (clone; 17A2), APC labeled CD4 (clone; RM4-5), PE labeled CD8 (clone; 53-6.7), APC-Cy7 labeled CD45R/B220 (clone; RA3-6B2). All antibodies were purchased from BD Biosciences. The expression of cell surface antigens was analyzed by flow cytometry (CyAn ADP, Beckman Colter Inc. or FACSVerse, BD Biosciences).

In order to check target-dependent T cell–activation, the expression levels of activation markers were analyzed by flow cytometry (BD FACSVerse) after *in vitro* tumor cell killing assay. These T-cells were stained with the following monoclonal antibodies: BUV395 labeled anti-mouse CD45 (BD Horizon, Clone; 30-F11), PE-CF594 labeled CD4 (BD Horizon, clone; RM4-5), perCP-Cy5.5 labeled CD8 (BD Horizon, clone; 53-6.7), FITC labeled CD25 (BD Pharmingen, clone; 7D4), APC/Cy7 labeled CD69 (BD Pharmingen, clone; H1.2F3).

#### Splenocyte proliferation and cytokine production

Splenocytes were cultured in complete RPMI1640 medium (Thermo Fisher) containing 10% fetal bovine serum and supplemented with PHA or LPS. These mitogens were purchased from SIGMA. For splenic T cell–stimulation via CD3, splenocyte cells were cultured on a plate coated with antibody to mouse Cd3 (145-2C11) or antibody to human CD3 (HIT3a). These antibodies were purchased from BD Biosciences. Two or 3 days after stimulation, splenocyte proliferation was analyzed by MTS assay (Promega) and culture supernatants were analyzed for cytokine concentrations using Cytometric Bead Array (BD Biosciences).

#### Immunization with an external antigen, OVA

An emulsion prepared by mixing chicken OVA and Freund’s complete adjuvant (OVA/FCA emulsion) was used for first immunization. Each mouse was subcutaneously injected with the OVA/FCA emulsion. Four weeks later, each mouse was immunized with OVA again, similarly to the first immunization except for using Freund’s incomplete adjuvant. One week after the second immunization, whole blood samples were collected from the inferior vena cava of the mice under isoflurane anesthesia. Serum levels of the OVA-specific immunoglobulin (Ig) E and Ig G1 were determined using the DS Mouse IgE ELISA (OVA) kit (DS Pharma, Japan) and Mouse OVA-IgG1 ELISA kit (Shibayagi, AKRIE-040, Japan), respectively, according to the manufacturer’s protocols.

#### Antibodies

A bispecific antibody to HER2 and hCD3 was generated by Fab arm exchange using anti-HER2 antibody (trastuzumab) and anti-human CD3 antibody (UCHT1). Bispecific antibody against hGPC3 and hCD3 was manufactured as full length human IgG4 by applying technologies for light chain commonization, heavy chain heterodimerization, and isoelectric point modification [Ishiguro, T. *et al*. Manuscript submitted]. Bispecific antibody against hGPC3 and mCD3 was generated by Fab arm exchange using anti-GPC3 antibody (GC33) and anti-mouse CD3 antibody (2C11). Anti-mouse PD-L1 antibody was purchased from BioXcell (clone: 10F.9G2).

#### Cells and culture conditions

Hepa1-6/hGPC3/HER2 cells (ATCC), which overexpressed hGPC3 and HER2 in Hepa1-6, were cultured in RPMI1640 (SIGMA) supplemented with 10% FBS (SIGMA), 0.6 mg/mL G418 (Nacalai Tesque), and 0.5 mg/mL Zeocin (Nacalai Tesque).

#### *In vitro* cytotoxicity and analysis of T-cell activation

*In vitro* cytotoxicity assays (TDCC) were monitored by xCELLigence Real-Time Cell Analysis (RTCA) system (ACEA Biosciences). The target cells (2.5 × 10^3^ cells) and spleen cells (1 × 10^5^ cells) from human *CD3 EDG*–replaced mice and wild-type (C57BL/6) mice were mixed with or without TRAB for 72 hours. The percentage of cytotoxicity was calculated as follows:


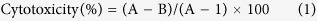


where A was the mean cell index of wells without TRAB; B was the mean cell index of wells with TRAB. Baseline was normalized as 1.

At the end of incubation, the cells were collected from the plate. Cells were stained with mouse CD4 (BD Horizon), CD8, CD25, CD69, CD152 (BD Pharmingen), and CD278 (eBioscience) and analyzed by flow cytometer.

#### *In vivo* models

The effects of TRABs or anti-PD-L1 antibody^34^ were tested in human *CD3 EDG*–replaced mice. Dosing and monitoring were performed in accordance with guidelines from the Institutional Animal Care and Use Committee at Chugai Pharmaceutical, Co., Ltd. Human *CD3 EDG*–replaced mice were subcutaneously inoculated with 10 million cells. Five mg/kg TRABs or 10 mg/kg anti-PD-L1 antibody were administered by i.v. injection in the tail vein. TGI was calculated as follows:


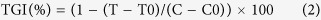


where T is tumor volume in the treated group at measurement, T0 is tumor volume in the treated group at baseline, C is tumor volume in the control group at measurement, and C0 is tumor volume in the control group at baseline.

#### Guidelines

Entire human *CD3 EDG*–replaced mice and human *CD3E* transgenic mice were generated in accordance with the Guidelines for the Care and Use of Laboratory Animals and the Ethical Guidelines for Research Using Human Specimens at Chugai Pharmaceutical Co. Ltd.

Protocols for the animal experiments, including generation of human *CD3 EDG*–replaced mice and human *CD3E* transgenic mice, RT-PCR for evaluation of transgenic human *CD3 EDG* and mouse endogenous *Cd3 edg* gene expression, immunohistochemical staining of CD3, flow cytometry analysis, splenocyte proliferation and cytokine production, immunization with an external antigen, OVA, *in vitro* cytotoxicity and analysis of T-cell activation, and *in vivo* models, were approved by the Care and Use of Laboratory Animals at Chugai Pharmaceutical Co. Ltd.

### Statistical analysis

Statistical analysis was performed using JMP 9.02 (SAS Institute Japan, Tokyo, Japan). Statistical significance in thymus and spleen weights were determined by Tukey-Kramer’s HSD test and MTS values were determined by nonparametric comparisons with control using the Dunn method for joint ranking. Tumor growth inhibition data was determined by Wilcoxon test. P < 0.05 was regarded as statistically significant.

## Additional Information

**How to cite this article:** Ueda, O. *et al*. Entire CD3ε, δ, and γ humanized mouse to evaluate human CD3–mediated therapeutics. *Sci. Rep.* **7**, 45839; doi: 10.1038/srep45839 (2017).

**Publisher's note:** Springer Nature remains neutral with regard to jurisdictional claims in published maps and institutional affiliations.

## Supplementary Material

Supplementary Information

## Figures and Tables

**Table 1:** Results of immunohistochemical staining for CD3 in mouse tissues.

Organs	Line	h*CD3 EDG*–replaced			h*CD3E* Tg	m*Cd3 edg*−/−	C57BL/6N
		4HH3	8I12	1C3	301	−	−
	Number of animals	3	3	3	3	3	3
Spleen	Lymphocyte, PALS	+++	+++	+++	+++	−	+++
	Lymphocyte, follicle	+	+	+	+	−	+
	Lymphocyte, red pulp	++	++	++	++	−	++
Thymus	Lymphocyte, cortex	+++	+++	+++	+++	−	+++
	Lymphocyte, medulla	+++	+++	+++	+++	−	+++
Mesenteric lymph node	Lymphocyte, paracortex	++	+++	+++	NA	−	+++
	Lymphocyte, follicle	+	+	+	NA	−	+
	Lymphocyte, medulla	+	+	+	NA	−	+
Ileum	Lymphocyte, GALT	+	++	++	NA	−	++
	Lymphocyte, lamina propria	+	+	+	NA	−	+
Other tissues^1)^	Lymphocyte	+	+	+	NA	−	+

IHC staining categories: −, negative; ±, rare;+, occasional;++, frequent;+++, constant. NA, not analysed.

^1)^Other tissues: liver, kidney, lung, heart, and adrenal gland.
