# Corrigendum: Entire CD3ε, δ, and γ humanized mouse to evaluate human CD3–mediated therapeutics

**DOI:** 10.1038/srep46960

**Published:** 2018-03-19

**Authors:** Otoya Ueda, Naoko A. Wada, Yasuko Kinoshita, Hiroshi Hino, Mami Kakefuda, Tsuneo Ito, Etsuko Fujii, Mizuho Noguchi, Kiyoharu Sato, Masahiro Morita, Hiromi Tateishi, Kaoru Matsumoto, Chisato Goto, Yosuke Kawase, Atsuhiko Kato, Kunihiro Hattori, Junichi Nezu, Takahiro Ishiguro, Kou-ichi Jishage

Correction to: Scientific Reports 7: Article number: 4583910.1038/srep45839; published online: 04 03 2017; updated: 03 19 2018

In this Article, the Authors neglected to include the Data and Material Availability section. This section should read:

“Data and material availability

The novel mouse strain generated in this study is available on request from Chugai Pharmaceutical Co., Ltd. (Chugai) under an MTA between the institution and Chugai. The following restrictions, in principle, apply to this mouse strain:

1. Cross-breeding the mouse strain with other mouse strains is prohibited.

2. Additional gene manipulations to the mouse strain are prohibited.

3. Use of the mouse strain for commercial purposes is prohibited.

4. Further transfer of the mouse strain to third parties is prohibited.

Our license agreement with GeneBridges restricts distribution of the BAC vectors, which were constructed using their Red/ET system.”

