# Supplementary material for: Entire CD3ε, δ, and γ humanized mouse to evaluate human CD3–mediated therapeutics
Source: Sci Rep. 2017 Apr 3;7:45839. doi: 10.1038/srep45839 (PMC5377452; doi:10.1038/srep45839)
Supplement: Supplementary Information [file srep45839-s1.doc]

Supplemental information for

Entire CD3ε, δ, and γ humanized mouse to evaluate human CD3–mediated therapeutics

**Authors:** Otoya Ueda^1^*, Naoko A. Wada^1^, Yasuko Kinoshita^2^, Hiroshi Hino^1^, Mami Kakefuda^3^, Tsuneo Ito^1^, Etsuko Fujii^1^, Mizuho Noguchi^2^, Kiyoharu Satoh^3^, Masahiro Morita^3^, Hiromi Tateishi^3^, Kaoru Matsumoto^3^, Chisato Goto^3^, Yosuke Kawase^3^, Atsuhiko Kato^1^, Kunihiro Hattori^2^, Junichi Nezu^4^, Takahiro Ishiguro^5^ and Kou-ichi Jishage^1^*

**Affiliations:**

^1^Chugai Pharmaceutical Co. Ltd., Research Division, Fuji Gotemba Research Labs., 1-135, Komakado, Gotemba, Shizuoka, Japan.

^2^ Chugai Pharmaceutical Co. Ltd., Research Division, Kamakura Research Labs., 200, Kajiwara, Kamakura, Kanagawa, Japan.

^3^ Chugai Research Institute for Medical Science Inc. 1-135, Komakado, Gotemba, Shizuoka, Japan.

^4^ Chugai Pharmabody Research Pte. Ltd., 3 Biopolis Drive, #07 - 11 to 16, Synapse, Singapore 138623.

^5^ Chugai Pharmaceutical Co. Ltd., Translational Clinical Research Science & Strategy Dept., 2-1-1, Muromachi, Nihonbashi, Chuou-ku, Tokyo, Japan.

*To whom correspondence should be addressed: jishagekui@chugai-pharm.co.jp and uedaoty@chugai-pharm.co.jp

| **Supplementary Table S1.** The protein accession numbers and the URL addresses for human and mouse CD3ε, CD3δ, and CD3γ | | |
| --- | --- | --- |
|  |  |  |
| **Protein** | **Accession#** | **URL address** |
| Human CD3E | NP_000724.1 | https://www.ncbi.nlm.nih.gov/protein/NP_000724.1 |
| Human CD3D | NP_000723.1 | https://www.ncbi.nlm.nih.gov/protein/NP_000723.1 |
| Human CD3G | NP_000064.1 | https://www.ncbi.nlm.nih.gov/protein/NP_000064.1 |
| Mouse Cd3e | NP_031674.1 | https://www.ncbi.nlm.nih.gov/protein/NP_031674.1 |
| Mouse Cd3d | NP_038515.3 | https://www.ncbi.nlm.nih.gov/protein/NP_038515.3 |
| Mouse Cd3g | NP_033980.1 | https://www.ncbi.nlm.nih.gov/protein/NP_033980.1 |

| **Supplementary Table S2.** A list of human CD3 transgenic mice established in this study | | | | | | | | |
| --- | --- | --- | --- | --- | --- | --- | --- | --- |
|  | | | | | | | | |
|  |  | Mouse | | |  | Human | | |
| Name of transgenic mice | Line# | *Cd3e* | *Cd3d* | *Cd3g* |  | *CD3E* | *CD3D* | *CD3G* |
| h*CD3 EDG*–replaced mice | 1C3* | KO^1)^ | KO | KO |  | Yes^3)^ | Yes | Yes |
| h*CD3 EDG*–replaced mice | 8I12 | KO | KO | KO |  | Yes | Yes | Yes |
| h*CD3 EDG*–replaced mice | 4HH3 | KO | KO | KO |  | Yes | Yes | Yes |
| h*CD3 EDG*–replaced mice | 2A4 | KO | KO | KO |  | ND.^4)^ | ND. | ND. |
| h*CD3 EDG*–replaced mice | 3B1 | KO | KO | KO |  | ND. | ND. | ND. |
| m*Cd3 edg^-/-^*** | - | KO | KO | KO |  | No^5)^ | No | No |
| h*CD3E* Tg mice | 301*** | Wild^2)^ | Wild | Wild |  | Yes | No | No |
| ^1)^ KO, knocked out allele. ^2)^ Wild, wild allele. ^3)^ Yes, Tg expression was detected. | | | | | | | | |
| ^4)^ ND., Tg expression was not detected. ^5)^ No, transgene was not introduced. | | | | | | | | |
| *, Official strain name: C57BL/6N-Del(9Cd3e-Cd3g)1Csk-Tg(CD3E,CD3D,CD3G)1Csk | | | | | | | | |
| **, Official strain name: C57BL/6N-Del(9Cd3e-Cd3g)1Csk | | | | | | | | |
| ***, Official strain name: C57BL/6N-Tg(hCD3E)301Csk | | | | | | | | |

| **Supplementary Table S3. Primers for genotyping by genomic PCR.** | | |
| --- | --- | --- |
| **Alleles** | **Forward primer** | **Reverse primer** |
| m*Cd3 edg*  (wild allele) | d168F  5’-AGATACAAGTGACCGAATATGAGG-3’ | d651R  5’-TTGCTATGGCACTTTGAGAAACCTCCATC-3’ |
| m*Cd3 edg-/-* | e30230F  5’-TAGCAGCCTTCAGATGAAGAGGTAGGACTC-3’ | g1439R  5’-TTGATGTGCCACCTCACTGCTGCACTGG-3’ |
| h*CD3 EDG* Transgene | hCD3e 5arm F2  5’-AACTGACAATGGGACATCAGCTGA-3’ | hCD3e ex2 R2  5’-ATGGGACTGTTACTTTACTAAGA-3’ |

| **Supplementary Table S4. Primers for detection of human *CD3 EDG* or mouse *Cd3 edg* expression by RT-PCR** | | | |
| --- | --- | --- | --- |
|  | **Gene** | **Forward primer** | **Reverse primer** |
| Human | *CD3E* | 5’-AAGAAATGGGTGGTATTACACAGACACC-3’ | 5’-TGGGCCAGCGGGAGGCAGTGTTCTCCAGAGG-3’ |
|  | *CD3D* | 5’-TAGTTCGGTGACCTGGCTTTATCTACTGG-3’ | 5’-ATGGCTGCTTCTAGAAGCCACCAGTCTCAGG-3’ |
|  | *CD3G* | 5’-TGCTCCACGCTTTTGCCGGAGGACAG-3’ | 5’-TAGGAGGAGAACACCTGGACTACTC-3’ |
|  | | | |
| Mouse | *Cd3e* | 5’-AGCATTCTGAGAGGATGCGGTGGAACAC-3’ | 5’-TGCTCGGAGGGCTGGATCTGGGTCCACAG-3’ |
|  | *Cd3d* | 5’-TCATCCTGTGGCTTGCCTCTATTTGTTGC-3’ | 5’-TTGCTATGGCACTTTGAGAAACCTCCATC-3’ |
|  | *Cd3g* | 5’-AATACTTCTACTGGAGAAGCAAAGAG-3’ | 5’-TAGTTGCATTTAGAGGACTTATTATGC-3’ |

| **Supplementary Table S5A.** Results of Tukey-Kramer's HSD tests for all combinations of genotypes in mouse spleen weights. | | | | | | | | |
| --- | --- | --- | --- | --- | --- | --- | --- | --- |
| Genotypes | Average (% of BW.) | S.D. | *p* values | | | | | |
|  |  |  | wt | m*Cd3 edg*  -/- | human *CD3 EDG*–replaced | | | h*CD3E* Tg |
|  |  |  |  |  | #1C3 | #4HH3 | #8I12 |  |
| wt | 0.303 | 0.005 | - | 0.4762 | 0.9995 | 0.3697 | 0.9997 | 0.4249 |
| m*Cd3 edg* -/- | 0.357 | 0.071 | 0.4762 | - | 0.3113 | **0.0128** | 0.3258 | **0.0160** |
| h*CD3 EDG*–replaced #1C3 | 0.293 | 0.052 | 0.9995 | 0.3113 | - | 0.5472 | 1.0000 | 0.6099 |
| h*CD3 EDG*–replaced #4HH3 | 0.243 | 0.026 | 0.3697 | **0.0128** | 0.5472 | - | 0.5286 | 1.0000 |
| h*CD3 EDG*–replaced #8I12 | 0.294 | 0.039 | 0.9997 | 0.3258 | 1.0000 | 0.5286 | - | 0.5911 |
| h*CD3E* Tg | 0.246 | 0.022 | 0.4249 | **0.0160** | 0.6099 | 1.0000 | 0.5911 | - |

Figures in blue denote statistically significant values.

| **Supplementary Table S5B.** Results of Tukey-Kramer's HSD tests for all combinations of genotypes in mouse thymus weights. | | | | | | | | |
| --- | --- | --- | --- | --- | --- | --- | --- | --- |
| Genotypes | Average  (% of BW.) | S.D. | *p* values | | | | | |
|  |  |  | wt | m*Cd3 edg* -/- | human *CD3 EDG*–replaced | | | h*CD3E* Tg |
|  |  |  |  |  | #1C3 | #4HH3 | #8I12 |  |
| wt | 0.310 | 0.048 | - | **<0.0001** | 0.7737 | **0.0100** | **0.0023** | **<0.0001** |
| m*Cd3 edg* -/- | 0.077 | 0.019 | **<0.0001** | - | **<0.0001** | **0.0075** | **0.0324** | 0.5384 |
| h*CD3 EDG*–replaced #1C3 | 0.272 | 0.015 | 0.7737 | **<0.0001** | - | 0.1364 | **0.0357** | **0.0011** |
| h*CD3 EDG*–replaced #4HH3 | 0.196 | 0.049 | **0.0100** | **0.0075** | 0.1364 | - | 0.9794 | 0.2153 |
| h*CD3 EDG*–replaced #8I12 | 0.175 | 0.060 | **0.0023** | **0.0324** | **0.0357** | 0.9794 | - | 0.5624 |
| h*CD3E* Tg | 0.127 | 0.033 | **<0.0001** | 0.5384 | **0.0011** | 0.2153 | 0.5624 | - |

Figures in blue denote statistically significant values

**
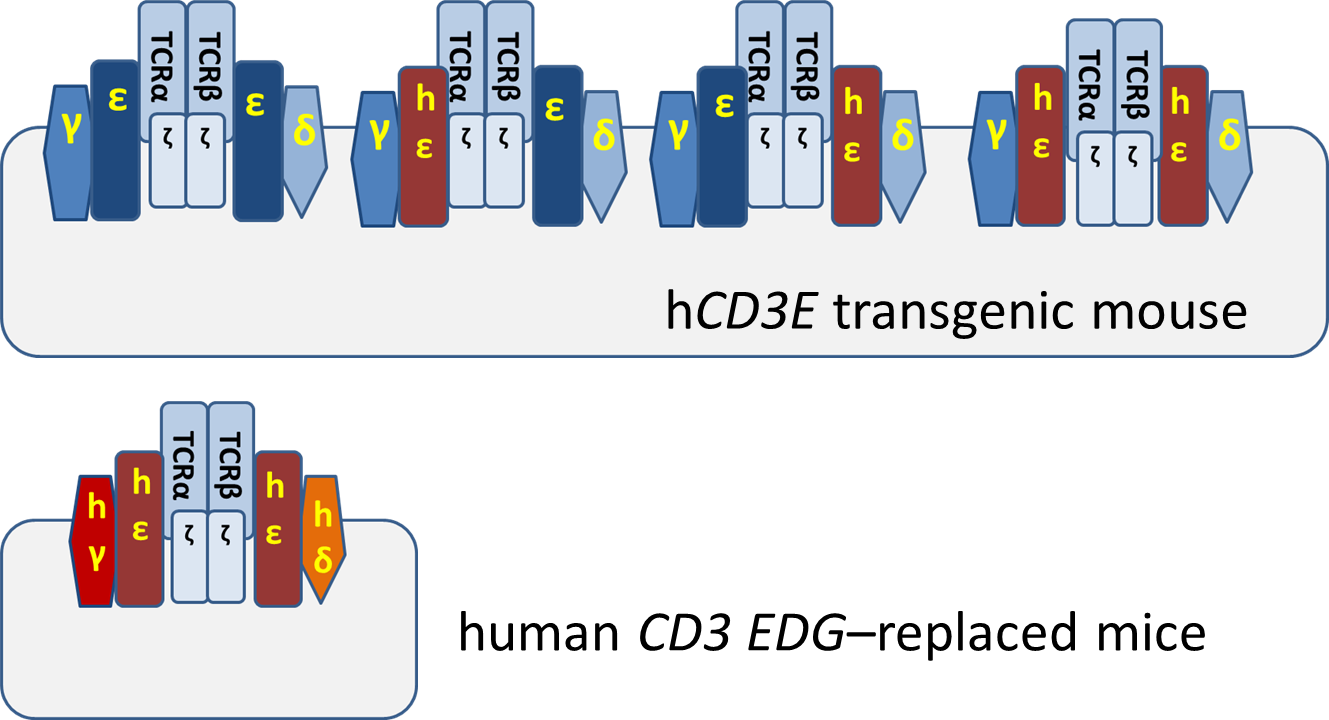
**

**Supplementary Figure S1.** A comparison of TCR-CD3 complex structures between human *CD3E* transgenic mice and human *CD3 EDG*–replaced mice. Co-localization of human CD3E or a more complicated TCR-CD3 complex formation could affect T cell development/differentiation in human *CD3E* transgenic mice. The TCR-CD3 complex structure is simple in human *CD3 EDG*–replaced mice.

**A**


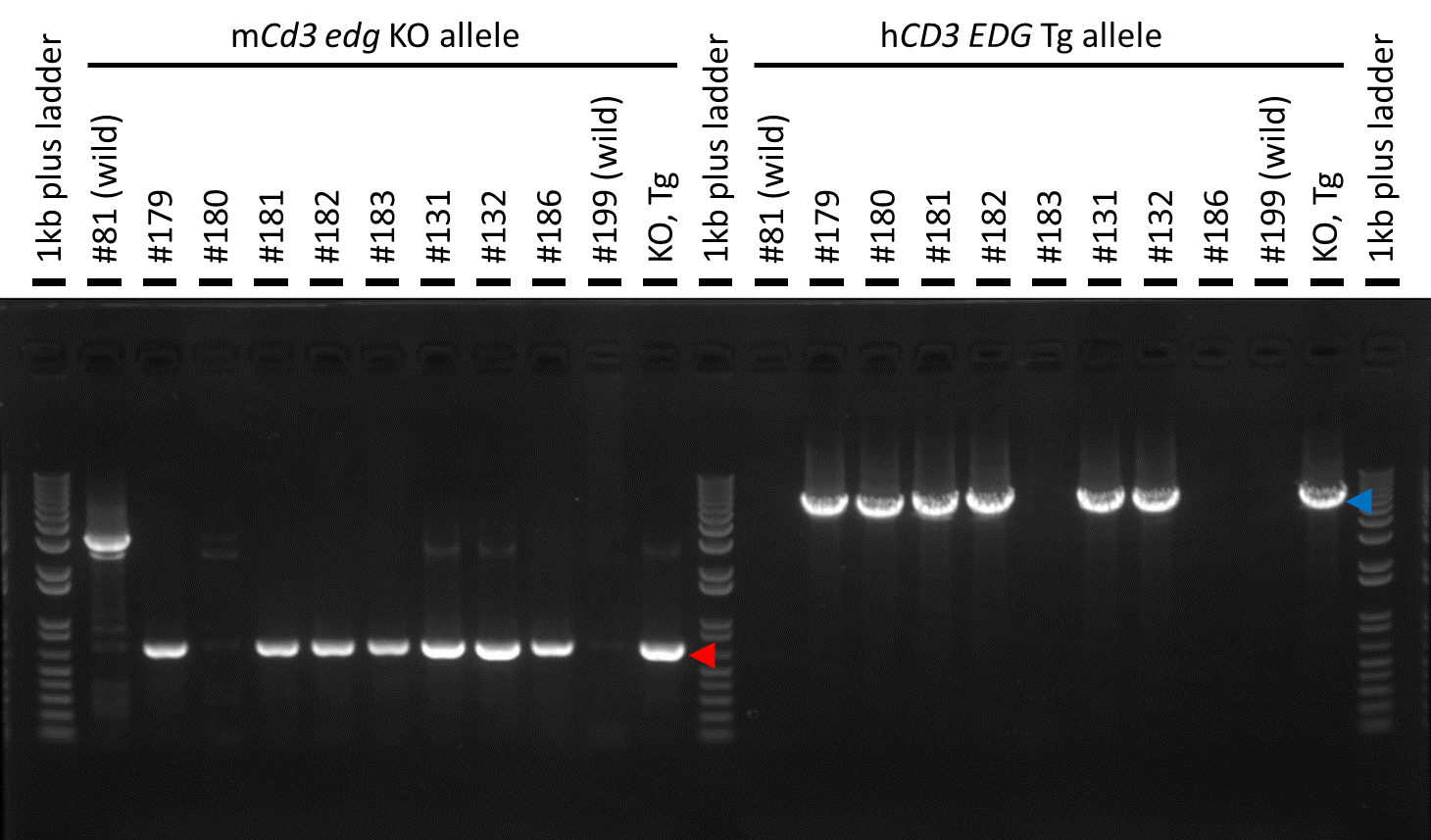


**B**


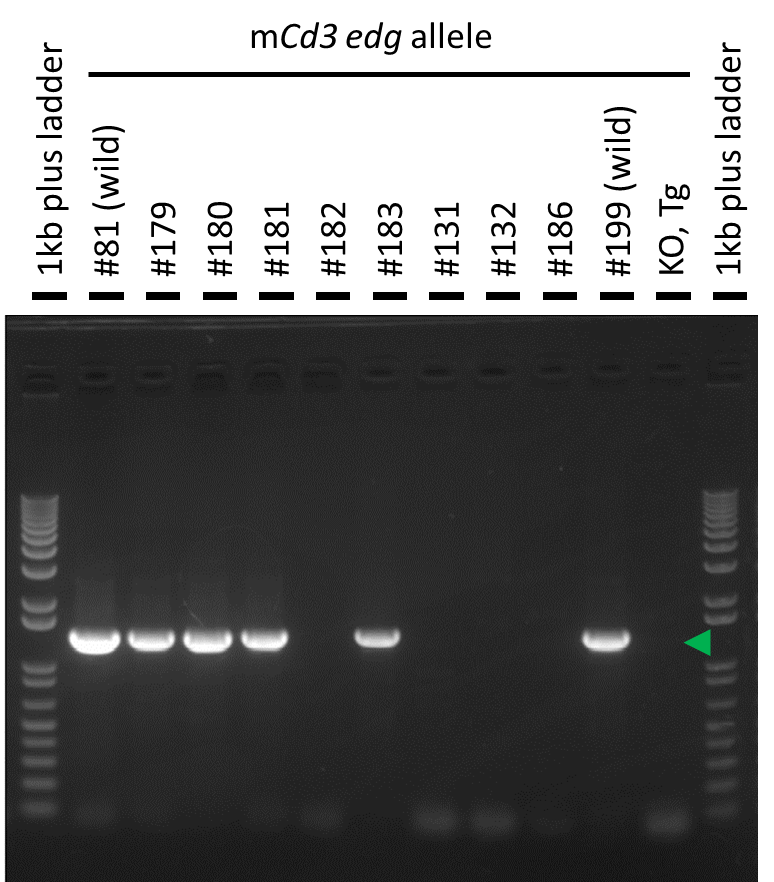


**Supplementary Figure S2A & S2B**

**Supplementary Figure 2.** Original gel electrophoresis images of representative genomic PCR for genotyping. Full images of PCR results on mouse *Cd3 edg* KO allele (**A**, left, signals indicated with red arrowhead), human *CD3 EDG* transgenic allele (**A**, right, signals indicated with blue arrowhead) and mouse *Cd3 edg* allele (**B,** signals indicated with green arrowhead) are shown. Number above each lane denotes the individual mouse number. Images shown in Figure 1C are the results of 6 mice on the left of each gel image (Mouse numbers: #81, #179, #180, #181, #182 and #183). KO, Tg: m*Cd3 edg-/-*; h*CD3 EDG* Tg: human *CD3 EDG*–replaced mice.


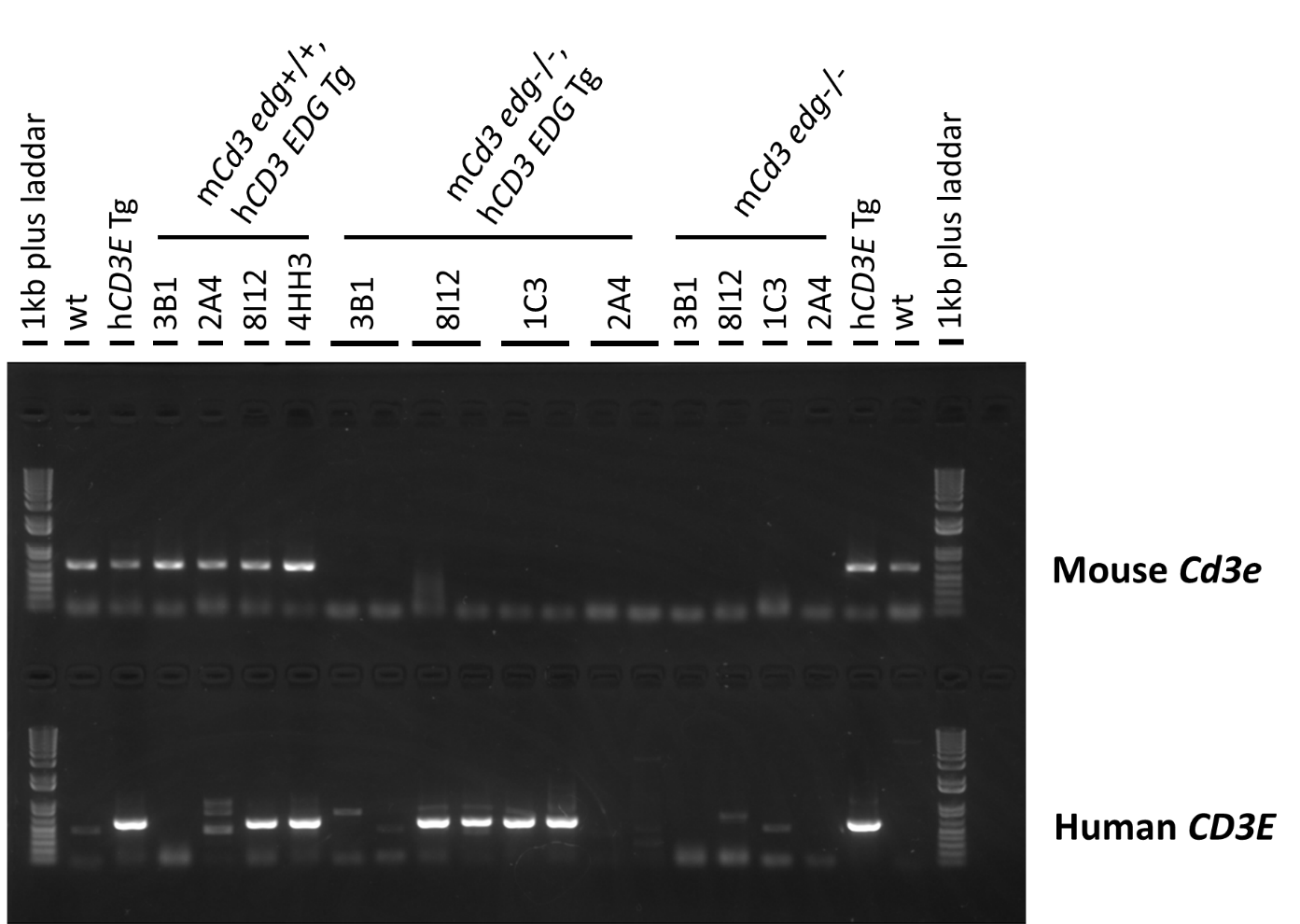

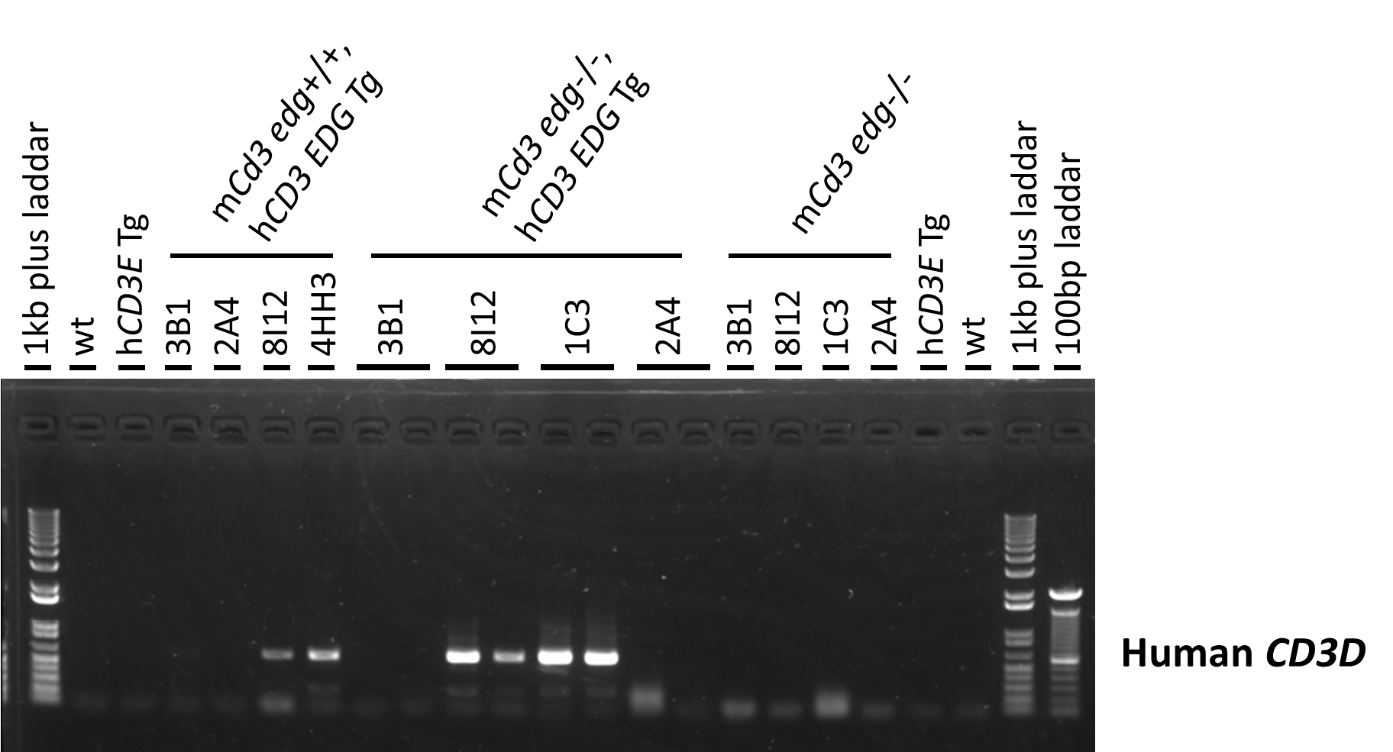


**B**

**A**

**Supplementary Figure S3A & S3B**

**C**


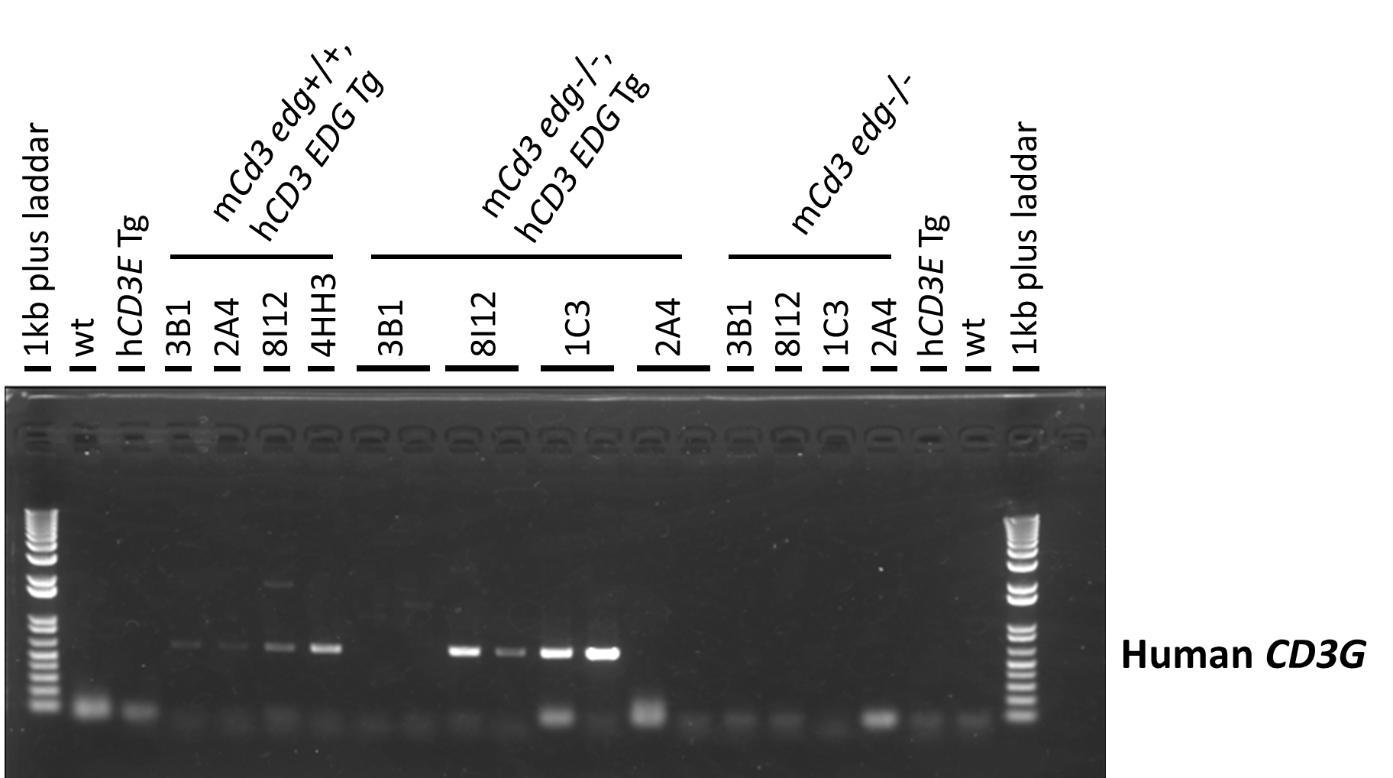


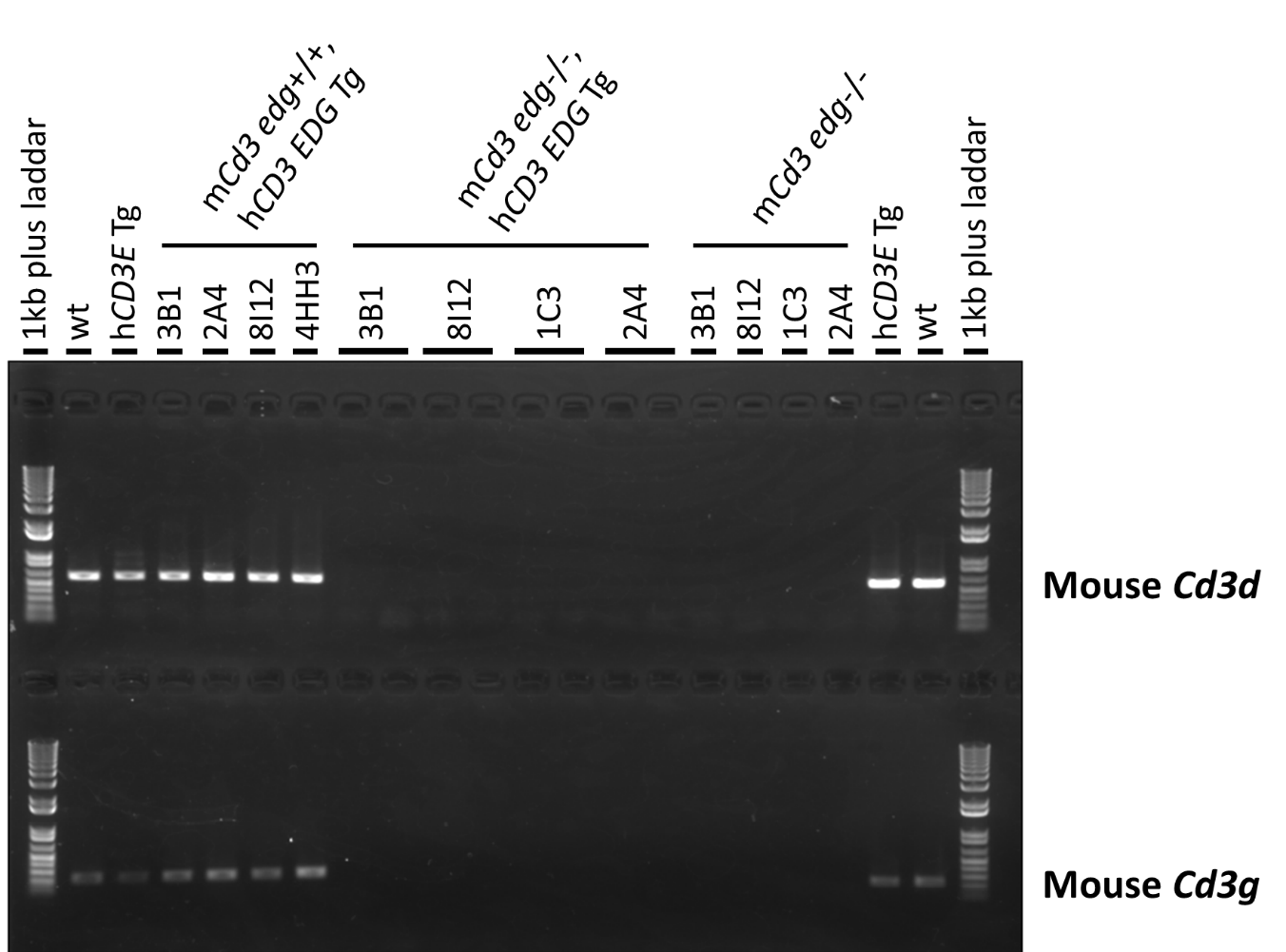


**D**

**Supplementary Figure S3C & S3D**

**E**


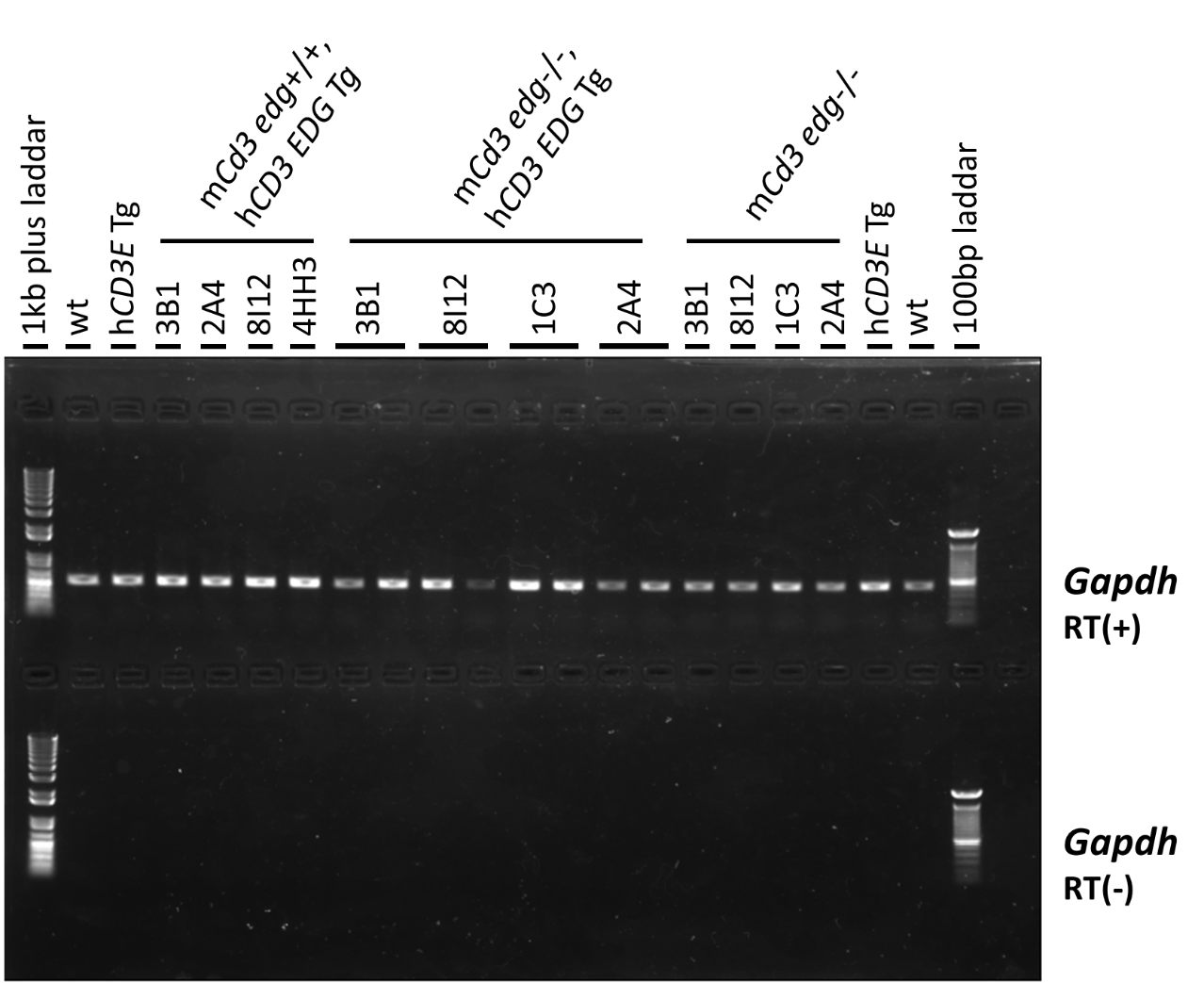


**Supplementary Figure S3.** Original gel electrophoresis images of the representative RT-PCR analysis in human *CD3 EDG*–replaced mice and the other controls. Full images are shown of (**A**) mouse *Cd3e* and human *CD3E*, (**B**) human *CD3D*, (**C**) human *CD3G*, and (**D**) mouse *Cd3d* and mouse *Cd3g*. (**E**) RT-PCR results of *Gapdh* with reverse transcription are shown in the upper row and the results of *Gapdh* without reverse transcription are shown in the lower row.


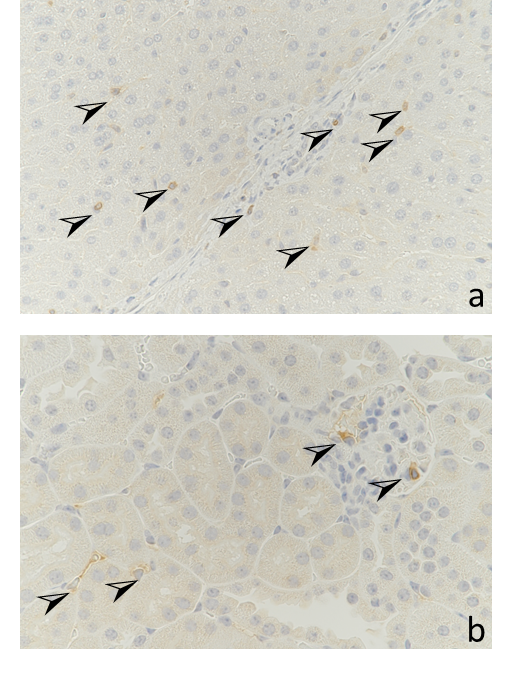


**Supplementary Figure S4.** Some CD3+ cells were observed in liver (a) and kidney (b). Arrowheads indicate CD3+ cells. These CD3+ cells were judged to be lymphocytes by their morphology.


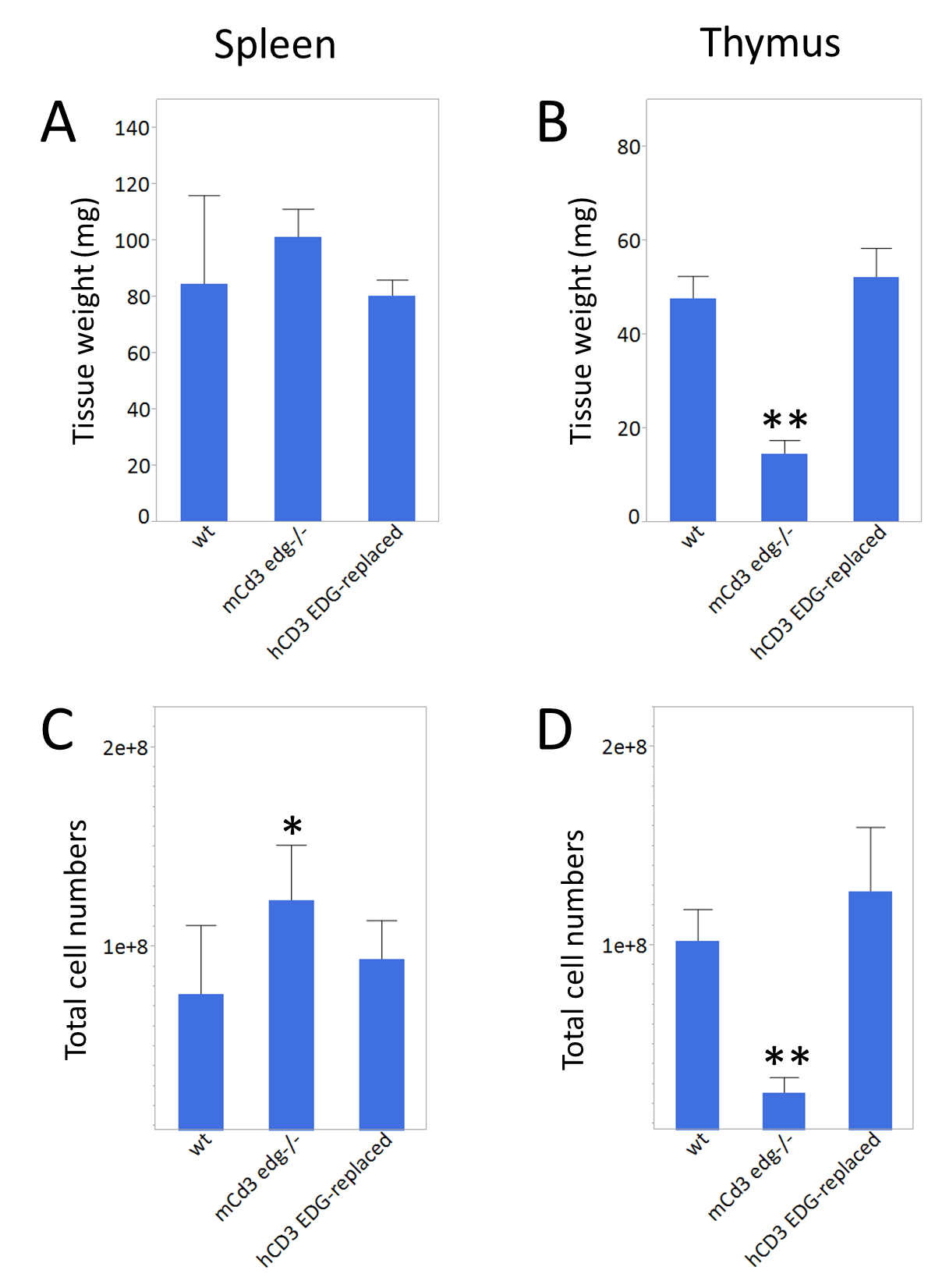


**Supplementary Figure S5.** The tissue weight of (**A**) spleen and (**B**) thymus and the total number of (**C**) splenocytes and (**D**) thymocytes in 4-month old male mice. Data are represented as mean ± s.d. (n=8, each genotype). Significant difference compared to wild-type mice. (*; P<0.05, **; P<0.0001 by Dunnett’s test for multiple comparisons.). wt, wild-type mice; m*Cd3 edg-/-*, mouse *Cd3 edg* knockout mice; hCD3 EDG-replaced, human *CD3 EDG*–replaced mouse line 1C3.

**
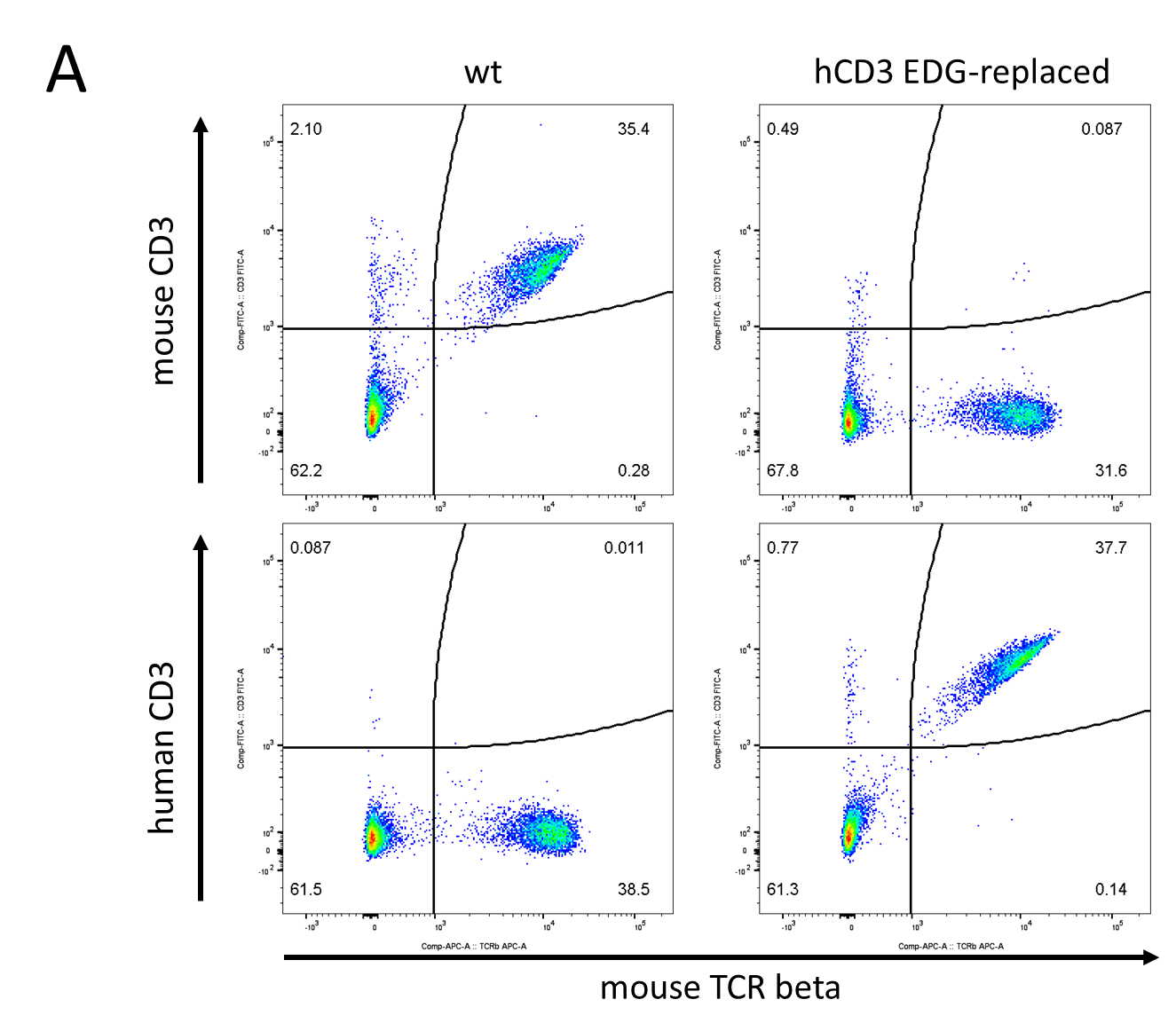
**

**Supplementary Figure S6A**


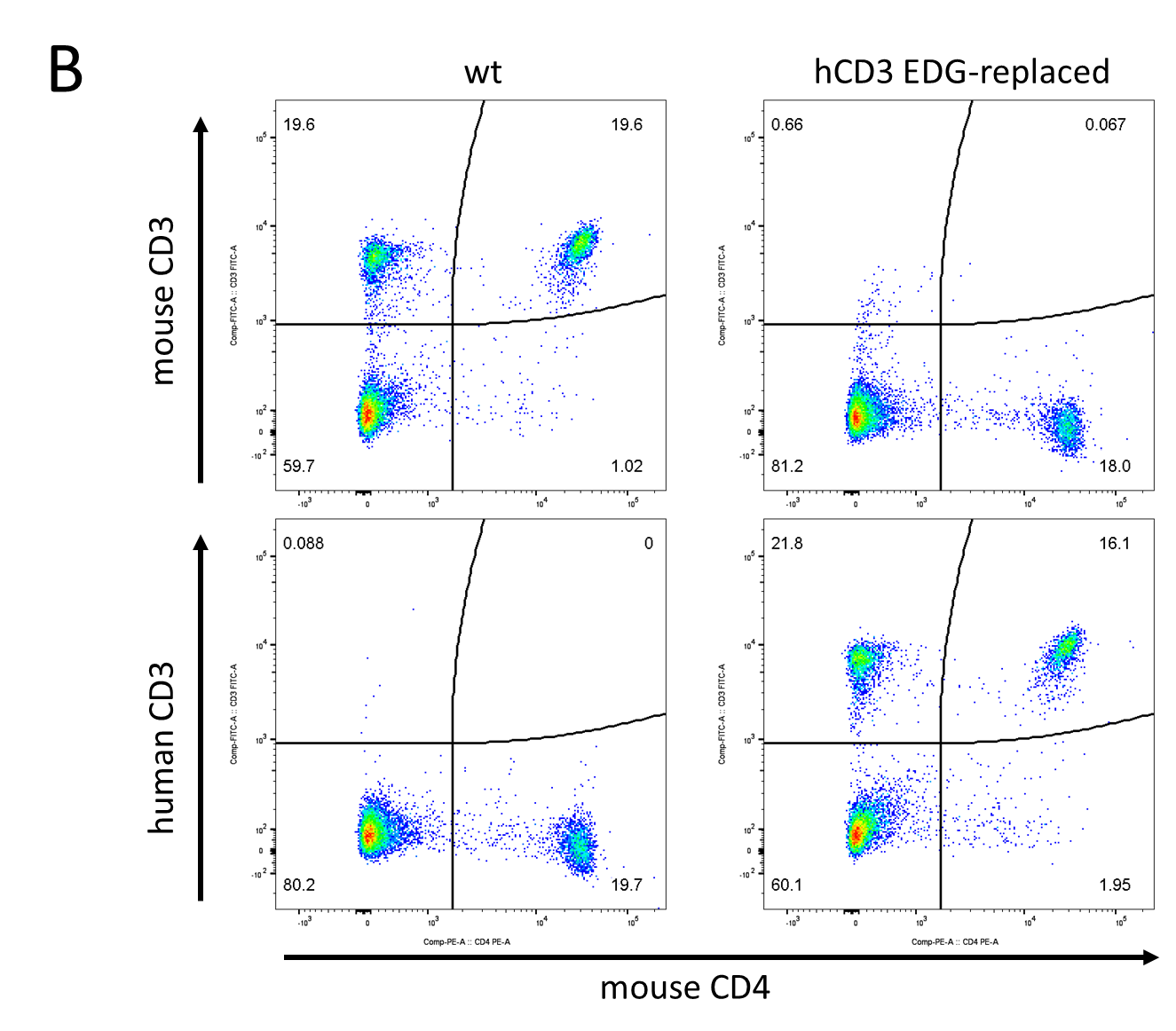


**Supplementary Figure S6B**


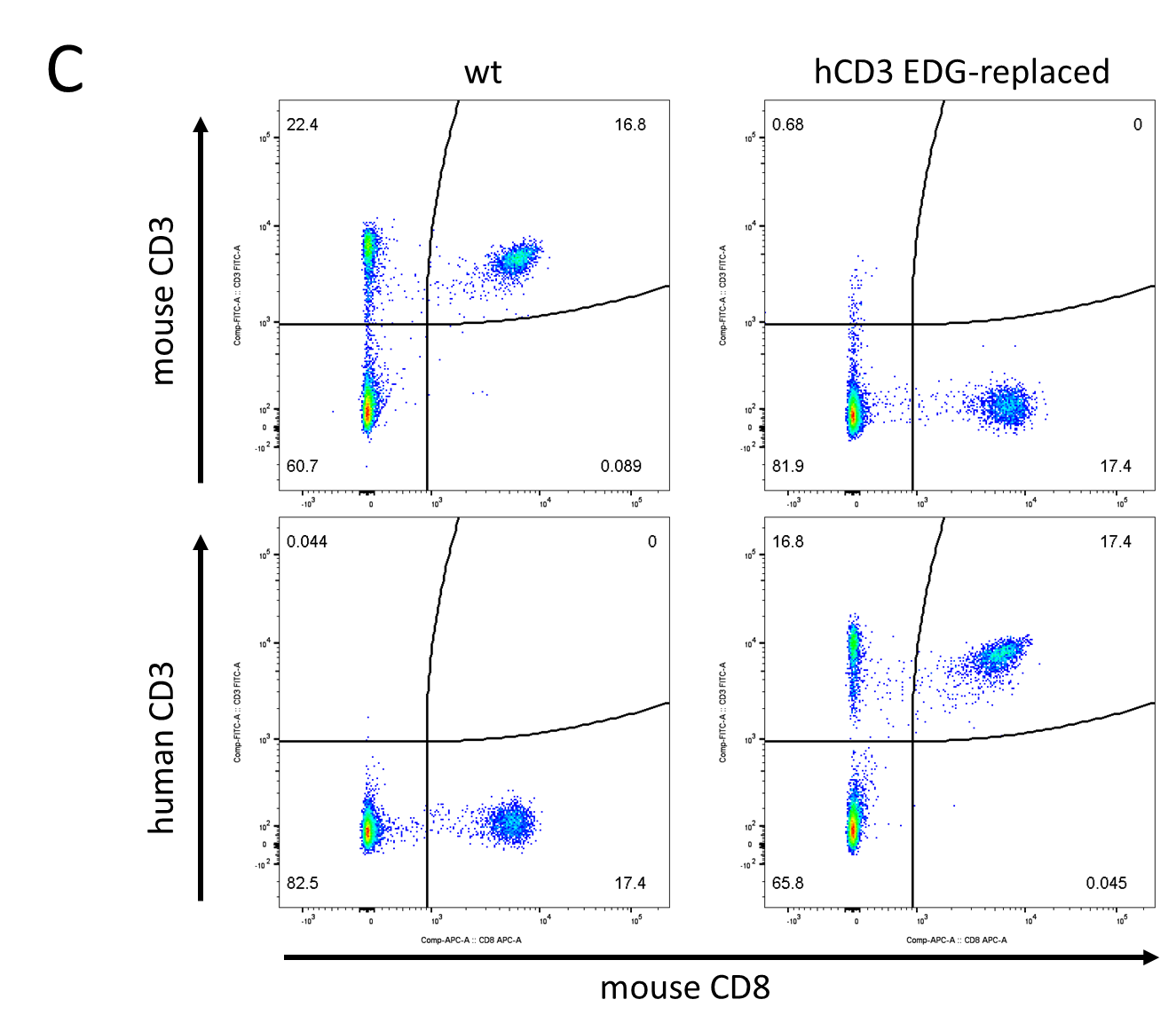


**Supplementary Figure S6C**

**Supplementary Figure S6. F**low cytometry was used to determine distribution in spleen of (**A**) CD3 positive and TCR beta positive cells, (**B**) CD3 positive and CD4 positive cells, and (**C**) CD3 positive and CD8 positive cells. Primary splenocytes were isolated from each mouse by mechanical dissociation followed by red blood–cell lysis. These splenocytes were stained with the following monoclonal antibodies: FITC labeled anti-human CD3 (clone; UCHT1), FITC labeled anti-mouse CD3 (clone; 17A2), PE labeled CD4 (clone; GK1.5), APC labeled CD8 (clone; 53-6.7), and APC labeled TCR beta (clone; H57-597). All antibodies were purchased from BD Biosciences. Representative cytograms of anti-mouse CD3 antibody (top) and anti-human CD3 antibody (bottom). wt, wild-type mice; hCD3 EDG-replaced, human *CD3 EDG*–replaced mouse line 1C3.
